# Single-cell analyses of intestinal epithelium reveal the dysregulation of gut immune microenvironment in systemic lupus erythematosus

**DOI:** 10.1186/s12967-025-06147-5

**Published:** 2025-01-27

**Authors:** Qiaolin Wang, Yutong Wu, Lianlian Ouyang, Xiaoli Min, Meiling Zheng, Lingyu Gao, Xiaoyun Chen, Zhi Hu, Shuang Yang, Wenjuan Jiang, Sujie Jia, Qianjin Lu, Ming Zhao

**Affiliations:** 1 Hospital for Skin Diseases, Institute of Dermatology, Chinese Academy of Medical Sciences and Peking Union Medical College, Nanjing, 210042 China; 2 Key Laboratory of Basic and Translational Research on Immune-Mediated Skin Diseases, Chinese Academy of Medical Sciences, Nanjing, 210042 China; 3 Department of Dermatology, Hunan Key Laboratory of Medical Epigenomics, Second Xiangya Hospital, Central South University, Changsha, 410011 China; 4 Department of Pharmacy, Chinese Academy of Medical Sciences and Peking Union Medical College, Nanjing, 210042 China

**Keywords:** Systemic lupus erythematosus, Gut immune environment, Intestinal intraepithelial lymphocyte, Enterocyte

## Abstract

**Background:**

The small intestine harbors a rich array of intestinal intraepithelial lymphocytes (IELs) that interact with structural cells to collectively sustain gut immune homeostasis. Dysregulation of gut immune homeostasis was implicated in the pathogenesis of multiple autoimmune diseases, however, whether this homeostasis is disrupted in a lupus autoimmune background remains unclear.

**Methods:**

We performed single-cell RNA sequencing (scRNA-seq) analyses to elucidate immune and structural milieu in the intestinal epithelium of MRL/Lpr lupus mice (Lpr mice) and MRL/Mpj control mice (Mpj mice). Comprehensive analyses including unsupervised clustering, trajectories, and cellular communication were performed. The primary findings from scRNA-seq were further validated by quantitative polymerase chain reaction (qPCR), flow cytometry, and in vivo experiments including selenium supplementation.

**Results:**

We observed a significant reduction in CD8αα + IELs, accompanied by a marked increase in CD8αβ + IELs in Lpr mice. Additionally, subsets of CD8 + IELs exhibiting significantly enhanced effector functions were found to be markedly enriched in Lpr mice. Intercellular communication patterns within intestinal epithelial immune and structural cells were found to be specifically altered in Lpr mice. Moreover, scRNA-seq revealed significantly decreased intestinal TCRγδ T cells (γδT) associated with reduced aryl-hydrocarbon receptor repressor (*AHRR*) expression and subsequent oxidative stress and ferroptosis in Lpr mice. Antioxidant selenium effectively reversed the loss of γδT in Lpr mice, improved the gut barrier, and alleviated lupus symptoms.

**Conclusions:**

Our high-resolution single-cell atlas enhances the understanding of the immune and structural milieu of intestinal epithelium in lupus and provides new insights into lupus pathogenesis mediated by intestinal immune dysregulation.

**Supplementary Information:**

The online version contains supplementary material available at 10.1186/s12967-025-06147-5.

## Introduction

The intestine is the largest immune organ in the human body in addition to its role in nutrient absorption [1]. The immune milieu in the gut is primarily constituted by intraepithelial immune cells, lamina propria immune cells, and gut-associated lymphoid tissues (GALTs) [2, 3]. Acting as sentinel lymphocytes, intestinal intraepithelial lymphocytes (IELs) play a critical role in immune surveillance and protection against pathogens by interacting with structural cells [4–6]. IELs also play a pivotal role in the pathogenesis of intestinal autoimmune disorders due to their involvement in upholding intestinal barrier integrity and maintaining immune tolerance [3]. Moreover, IELs have been implicated in the modulation of autoimmune responses in extra-intestinal diseases. One example of this modulation was the reported suppression of central nervous system autoimmunity by CD4 + T cells residing within the intestinal epithelium, mediated by lymphocyte-activation gene 3 (LAG-3) [7]. A detailed summary of the relationship between immune cells in the intestine and autoimmunity has been systematically presented in our previous reviews [8–10].

Systemic lupus erythematosus (SLE) is an autoimmune-mediated disease characterized by widespread damage to multiple organs and systems, with particular attention from the scientific community to the occurrence of SLE cutaneous lesions and renal impairment [11, 12]. Dysregulation of the gut microbiota is recognized to play a pivotal role in SLE pathogenesis. Our team’s clinical trials have shown that SLE patients who received fecal microbiota transplantation from healthy donors experienced significant symptom relief [13–15]. Furthermore, SLE is associated with notable disruption of the gut barrier, with microbial translocation secondary to compromised gut barrier implicated in SLE pathogenesis [16]. However, the mechanisms underlying the gut barrier disruption remain poorly understood. Dysbiosis of the gut microbiota commonly coincides with disturbances in the gut immune microenvironment [8, 17]. While extensive research has focused on gut microbiota dysbiosis in SLE, alterations in the gut immune microenvironment in SLE have been largely overlooked. Our previous findings demonstrated that SLE could significantly increase the risk of celiac disease, suggesting a possible dysregulation of the intestinal immune milieu in SLE [18]. In addition, the crosstalk between intestinal immune cells and structural cells is crucial for maintaining intestinal homeostasis [19], yet whether this crosstalk is disrupted in SLE remains uncertain. To fill the present knowledge lacunae, we conducted a study based on single-cell RNA sequencing (scRNA-seq) to decipher the immune and structural milieu in intestinal epithelium in.

MRL/Lpr lupus mice (Lpr mice), which develop spontaneous systemic autoimmunity with many hallmarks of the human disease systemic lupus erythematosus. Subsequently, flow cytometry analyses and in vivo experiments were used to validate the findings from scRNA-seq.

In the study, a total of 38,737 immune and structural cells isolated from the epithelium layers of the small intestine from three twenty-week-old Lpr mice and three age-matched control MRL/Mpj mice (Mpj mice) were further analyzed. Our analysis revealed heterogeneity in CD8+ IEL subsets and structural cells within the intestinal epithelium of Lpr mice. Additionally, crosstalk patterns within intestinal epithelial immune and structural cells were found to be specifically altered in Lpr mice. Furthermore, oxidative stress-associated γδT loss may contribute to the gut barrier disruption in Lpr mice. Supplementation with selenium, an antioxidant, effectively reversed intestinal γδT loss in Lpr mice, improved the gut barrier, and alleviated lupus symptoms. In summary, our high-resolution single-cell atlases advance the understanding of lupus intestinal immune microenvironment and provide new insights into lupus pathogenesis.

## Results

### scRNA-seq illustrated a high-resolution immune and structural landscape of the intestinal epithelial layer in lupus mice

**Fig. 1:**
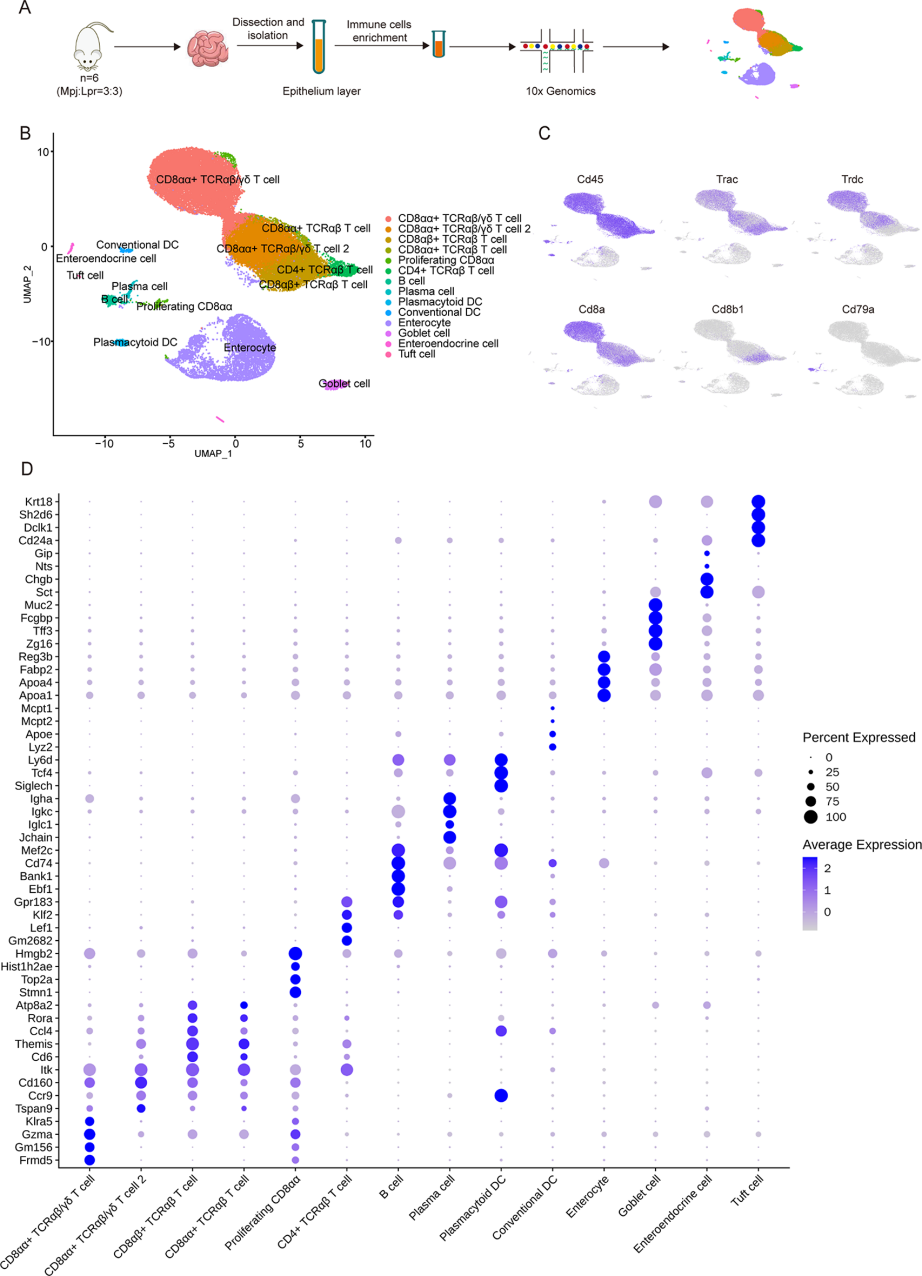
scRNA-seq illustrates a high-resolution immune and structural landscape of the intestinal epithelial layer in lupus mice. (**A**) Workflow of the experimental pipeline. (**B**) UMAP plot depicting main cell types identified in intestinal epithelial layer, based on a total of 38,737 cells. (**C**) Feature plots representing expression of *Cd45*,* Trac*,* Trdc*,* Cd8a*,* Cd8b1*, and *Cd79a*. (**D**) Dot plot illustrating the selected top DEGs for immune cells and structural cells in the intestinal epithelial layer. Color saturation denotes the strength of gene expression on average, while the size of dots corresponds to the proportion of each cell cluster expressing the gene. DC: Dendritic cells

To investigate the cell composition of the epithelium layer of the small intestine in lupus mice. We isolated the small intestine from three twenty-week-old Lpr mice and three age-matched Mpj mice, which served as controls for the Lpr mice. To increase the proportion of immune cells in scRNA-seq, single-cell suspensions were prepared by Percoll 40–80% gradient separation for immune cell enrichment after tissue dissection, followed by scRNA-seq based on the 10X Genomics system (Fig. 1A and Supplementary Table 1). A total of 38,737 cells were acquired after filtering low-quality cells and correcting batch effects (Supplementary Fig. 1A, B). Through uniform manifold approximation and projection (UMAP) clustering and the analysis of differential expression of hallmark genes, we revealed 10 immune cell clusters and 4 structural cell clusters in intestinal epithelium layer, including CD8αα + TCRαβ/γδ T cell, CD8αα + TCRαβ/γδ T cell 2, CD8αβ + TCRαβ cell, CD8αα + TCRαβ cell, proliferating CD8αα cell, CD4 + TCRαβ T cell, B cell, plasma cell, plasmacytoid dendritic cell (pDC), conventional dendritic cell (cDC), enterocyte, goblet cell, enteroendocrine cell, tuft cell (Fig. 1B and Supplementary Table 2). Immune cells were defined by high expression of *Cd45*, *Trac*, *Trdc*, *Cd8a*, *Cd8b1*, *Cd79a*, *Cd4*, and *Siglech*, while structural cells were defined by high expression of *Epcam*, *Muc2*, *Chga*, and *Dclk1* (Fig. 1B, C and Supplementary Fig. 1C). Of note, the CD8αα + T cells contained TCRγδ T cells (γδT) and TCRαβ T cells, implying high transcriptional similarities between γδT and TCRαβ T cells within the cluster. Therefore, the term “TCRαβ/γδ T cell” was used to indicate the cell populations that contain both γδT and TCRαβ T cells. Similar naming conventions in intestinal immune cells have also been reported in other literature [19]. TCRαβ/γδ T cells represented the predominant immune cell population in our scRNA-seq data, and they were further separated into two clusters based on the illustration by the UMAP plot. The top 4 differentially expressed genes (DEGs) for immune cells and structural cells are presented in Dot plot (Fig. 1D).

### Significantly altered CD8 + T cell compositions in the intestinal epithelial layer of lupus mice

**Fig. 2:**
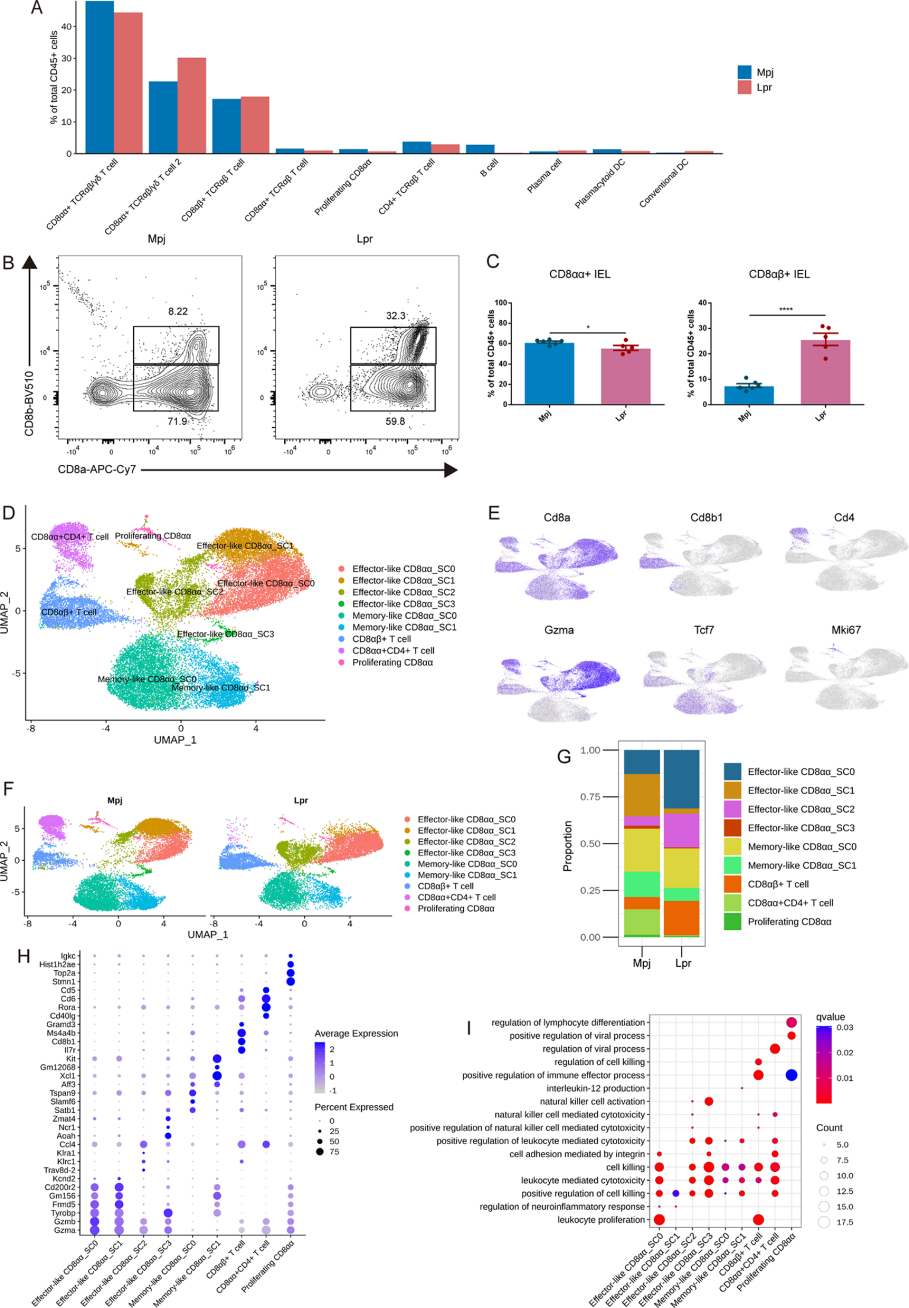
Significantly altered CD8 + T cell compositions in the intestinal epithelial layer of lupus mice. (**A**) Histogram showing the proportion of intestinal intraepithelial immune cells. (**B** and **C**) Flow cytometry analysis was conducted in a supplementary group of six Mpj mice and five Lpr mice to examine CD8 + T cell populations, gating on CD45 + CD3 + IELs. Each dot denotes an individual mouse. (**D**) UMAP plot of CD8 + T cells in the intestinal epithelial layer, based on a total of 29,236 cells. (**E**) Feature plots representing expression of *Cd8a*,* Cd8b1*,* Cd4*,* Gzma*,* Tcf7*, and *Mki67*. (**F** and **G**) UMAP plot of CD8 + T cell subsets (**F**) and frequency of CD8 + T cell subsets among total CD8 + T cells (**G**) in Mpj and Lpr mice. (**H**) Dot plot illustrating the selected top DEGs for the CD8 + T cell subsets. Color saturation denotes the strength of gene expression on average, while the size of dots corresponds to the proportion of each cell cluster expressing the gene. **(I)** Bubble diagrams display the Gene Ontology (GO) biological process (BP) terms that are enriched in each CD8 + T cell subset. Statistical significance in (**C**) was determined by student’s *t*-test. Each dot denotes an individual sample. *P < 0.05, **P < 0.01, ***P < 0.001, ****P < 0.0001

CD8 + T cells are the predominant immune cell population in intestinal epithelium layer [3]. scRNA-seq revealed that CD8αα + TCRαβ/γδ T cell subset was reduced while CD8αα + TCRαβ/γδ T cell 2 subset was increased in Lpr mice (Fig. 2A). Other increased immune cell subsets in Lpr mice included CD8αβ + TCRαβ cell, plasma cell, and cDC, while decreased subsets included CD8αα + TCRαβ cell, proliferating CD8αα cell, CD4 + TCRαβ T cell, B cell, pDC (Fig. 2A and Supplementary Fig. 2). Flow cytometry analyses were conducted in a supplementary group of 6 Mpj mice and 5 Lpr mice to examine the change of CD8 + T cell populations, and found that CD8 + IELs were significantly increased in total CD45 + immune cells (Supplementary Fig. 3A, B). Further analysis showed that the increased CD8 + IELs primarily consisted of CD8αβ + IELs while the proportion of CD8αα + IELs was markedly decreased (Fig. 2B, C), which was consistent with the result in scRNA-seq revealed that CD8αα + TCRαβ/γδ T cell, the most predominant immune cell population in CD8 + T cells, was significantly decreased in Lpr mice. To examine the potential cellular heterogeneity within CD8 + T cells in Lpr mice, a total of 29,236 CD8 + T cells were further analyzed. UMAP visualization unveiled nine distinct clusters (Fig. 2D, Supplementary Fig. 4A, B, and Supplementary Table 3). In addition to the identification of CD8 + T cells through the expression of Cd8a and Cd8b1, Cd4 expression within a subset of CD8αα + T cells suggested their classification as CD8αα + CD4 + T cells (Fig. 2E). Most CD8αα + T cells expressed cytotoxicity-associated gene *Gzma* or memory-associated gene *Tcf7*, then they were defined as effector-like CD8αα (4 clusters) or memory-like CD8αα (2 clusters). Stem cell-related gene *Mki67* expression within a subset of CD8αα + T cells suggested their classification as proliferating CD8αα cells. UMAP plot exhibited that effector-like CD8αα or memory-like CD8αα T cells were the primary subpopulations of all CD8 + T cells (Fig. 2D). Evaluation of the proportion of each CD8 + T cell subset revealed an increased proportion in total effector-like CD8αα and a decreased proportion in total memory-like CD8αα in Lpr mice (Fig. 2F, G and Supplementary Fig. 4C). SC0 and SC2 clusters were the main effector-like CD8αα subsets increased in Lpr mice (Fig. 2G). Top DEGs showed that effector-like CD8αα_SC0 contained the highest expression of *Gzma* and *Gzmb* (Fig. 2H), suggesting an enhanced effector function in the cluster compared to other effector-like CD8αα clusters.

Further Gene Ontology (GO) biological process (BP) analysis showed that positive regulation of cell killing, leukocyte mediated cytotoxicity was commonly enriched in effector-like CD8αα_SC0 and effector-like CD8αα_SC2 (Fig. 2I and Supplementary Table 4). GO BP terms associated with enhanced effector function such as cell killing and natural killer cell activation were also presented in effector-like CD8αα_SC3 while the cluster constitutes a small proportion. In addition, CD8αβ + T cell was also observed in an obvious expansion in Lpr mice and enriched BP terms including cell killing, regulation of cell killing, and positive regulation of immune effector process. Of note, a significantly decreased proportion in CD8αα + CD4 + T cells was found in Lpr mice, although the cluster also contained the function related to cell killing based on BP terms. Regulation of the viral process was mainly enriched in CD8αα + CD4 + T cells. In addition to CD8 + T cells, other immune cells in the intestinal epithelial layer constitute only a minor proportion, such as CD4 + IELs, B cells, plasma cells, and DC. The differential genes of these cells are presented in Supplementary Fig. 5A-E and Supplementary Tables 5–9.

These results suggested a significant alteration in the composition of intestinal epithelial CD8 + T cells in Lpr mice, manifested by a shift in proportion from memory-like CD8αα in Mpj mice to effector-like CD8αα in Lpr mice, and effector function was significantly enhanced in the expanded CD8αα and CD8αβ clusters within Lpr mice intestines.

### Altered functions of structural cells were revealed in lpr mice intestines

**Fig. 3:**
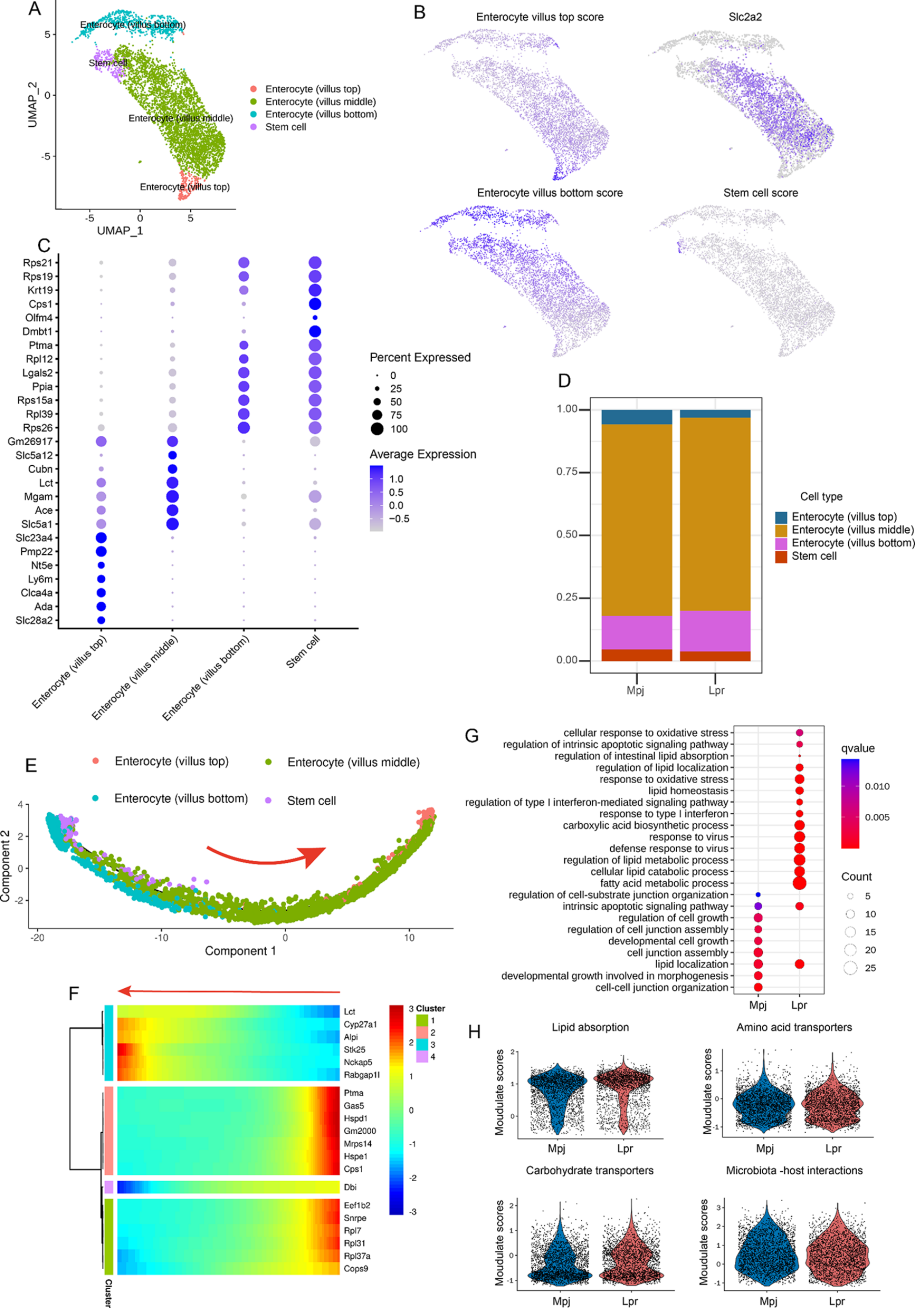
The composition of enterocytes and changed nutrient absorption in lupus mice intestines. (**A**) UMAP plot of enterocytes, based on a total of 4,908 cells. (**B**) Average scores of signature genes for cluster annotation of enterocyte*s* (genes indicated in supplementary Table 10). Average scores in each cell are indicated in color saturation. (**C**) Dot plot illustrating selected top DEGs for enterocyte subsets. Color saturation denotes the strength of gene expression on average, while the size of dots corresponds to the proportion of each cell cluster expressing the gene. (**D**) Histogram showing the proportions of enterocyte subsets. (**E**) Pseudotime trajectory analysis of enterocyte subtype is illustrated using trajectory plot. Distinct colors represent enterocyte subtypes. The arrow represents a possible differentiation direction. (**F**) Heatmap indicates gene clusters with the differentiation state that were predicted by pseudotime trajectory analysis. The arrow shows the possible differentiation direction. Color saturation denotes the strength of gene expression. (**G**) Bubble diagrams display the Gene Ontology (GO) biological process (BP) terms that are enriched in the total enterocytes of Mpj and Lpr mice. (**H**) Violin plots are utilized to depict the average signature scores calculated for lipid absorption, amino acid transporters, carbohydrate transporters, and microbiota-host interactions. Each dot represents a single cell

Intestinal epithelial structural cells and immune cells constitute the intestinal epithelium. Structural cells, such as enterocytes and goblet cells, play a critical role in gut barrier maintenance, the absorption of nutrients, and intestinal immune homeostasis [19]. Nonetheless, the modulation of intestinal epithelial structural cells in a lupus autoimmune background remains poorly elucidated. Histological examination revealed shortened villi in the ileum of Lpr mice, and the expression of zona occludens-1 (*Zo-1*), a tight junction protein, was down-regulated in the ileal tissue of Lpr mice (Supplementary Fig. 6A, B, C). To further investigate the heterogeneity of structural cells. Firstly, we obtained a total of 4,908 enterocytes in scRNA-seq to investigate its heterogeneity in Lpr mice intestines. In addition to stem cells, enterocytes were further divided into three clusters along the intestinal villus axis (Fig. 3A and Supplementary Fig. 7A, B) [20]. The enterocytes at the villus top and bottom were identified based on modulation scores, while the enterocytes in the villus middle were identified by the expression of Slc2a2 (Fig. 3B and Supplementary Table 10) [20, 21]. Top DEGs indicated distinct expression profiles within enterocyte clusters (Fig. 3C and Supplementary Table 11). Evaluation of the proportion of the clusters showed that the enterocytes in the middle represented the predominant subpopulation in total enterocytes (Fig. 3D and Supplementary Fig. 7C), and the proportions in each cluster were similar between Mpj mice and Lpr mice, while the enterocytes in the villus top exhibited a slight decrease in Lpr mice intestines. Subsequently, pseudotime trajectory analysis showed a potential differentiation trajectory from stem cell/enterocyte (villus bottom) to enterocyte (villus middle), and finally to enterocyte (villus top) (Fig. 3E, F).

Further GO BP analysis based on DEGs revealed that total enterocytes in Lpr mice enriched the terms associated with oxidative stress such as cellular response to oxidative stress, and the terms associated with type I interferon such as regulation of type I interferon-mediated signaling pathway, and response to type I interferon (Fig. 3G, Supplementary Fig. 7D, and Supplementary Tables 12, 13). SLE is characterized by widespread activation of the Type I interferon signaling pathway in immune cells [22]. The pathways enriched in enterocytes suggested a potential impact of lupus autoimmunity on the structural cells in the intestines. In addition, pathways related to lipid homeostasis were also significantly enriched in Lpr mice, such as regulation of lipid absorption, regulation of lipid localization, regulation of lipid metabolic process, and fatty acid metabolic process. Compared to Mpj mice, the pathways significantly downregulated in the Lpr mice included regulation of cell junction assembly, cell junction assembly, and cell-cell junction organization, suggesting an impaired gut barrier in Lpr mice. Leaky gut along with subsequent bacterial translocation constitutes one of the pivotal mechanisms underlying the pathogenesis of SLE [23]. Here, we also examined whether the nutrient absorption function of enterocytes was altered in the context of lupus, and found that lipid absorption modulation scores were enhanced in the enterocytes of Lpr mice (Fig. 3H and Supplementary Table 10), which was consistent with GO BP analysis results.

In addition to enterocytes, various types of structural cells exist within the intestinal epithelium. UMAP plot exhibited enteroendocrine cells, tuft cells, and goblet cells in our scRNA-seq. However, Paneth cells were not annotated in our data due to the limited number of cells. To investigate the heterogeneity of the structural cells, Top DEGs were identified between Mpj and Lpr mice (Supplementary Fig. 8A-C and Supplementary Tables 14–16). GO BP analysis showed that goblet cells from Lpr mice intestines enriched the terms related to inflammatory pathways such as response to interferon-gamma, positive regulation of cytokine production, and regulation of cytokine production involved in immune response (Supplementary Fig. 8D, Supplementary Table 17), suggesting a functional alteration of goblet cells in the context of lupus. Taken together, these findings indicated an altered function of structural cells in lupus mice intestines, particularly enterocytes, which were manifested primarily in enhanced lipid absorption, response to type I interferon, as well as the changes in cell junction-related pathways, suggesting the compromised integrity of the lupus gut barrier.

### A unique intestine signalome was revealed in lupus mice

**Fig. 4:**
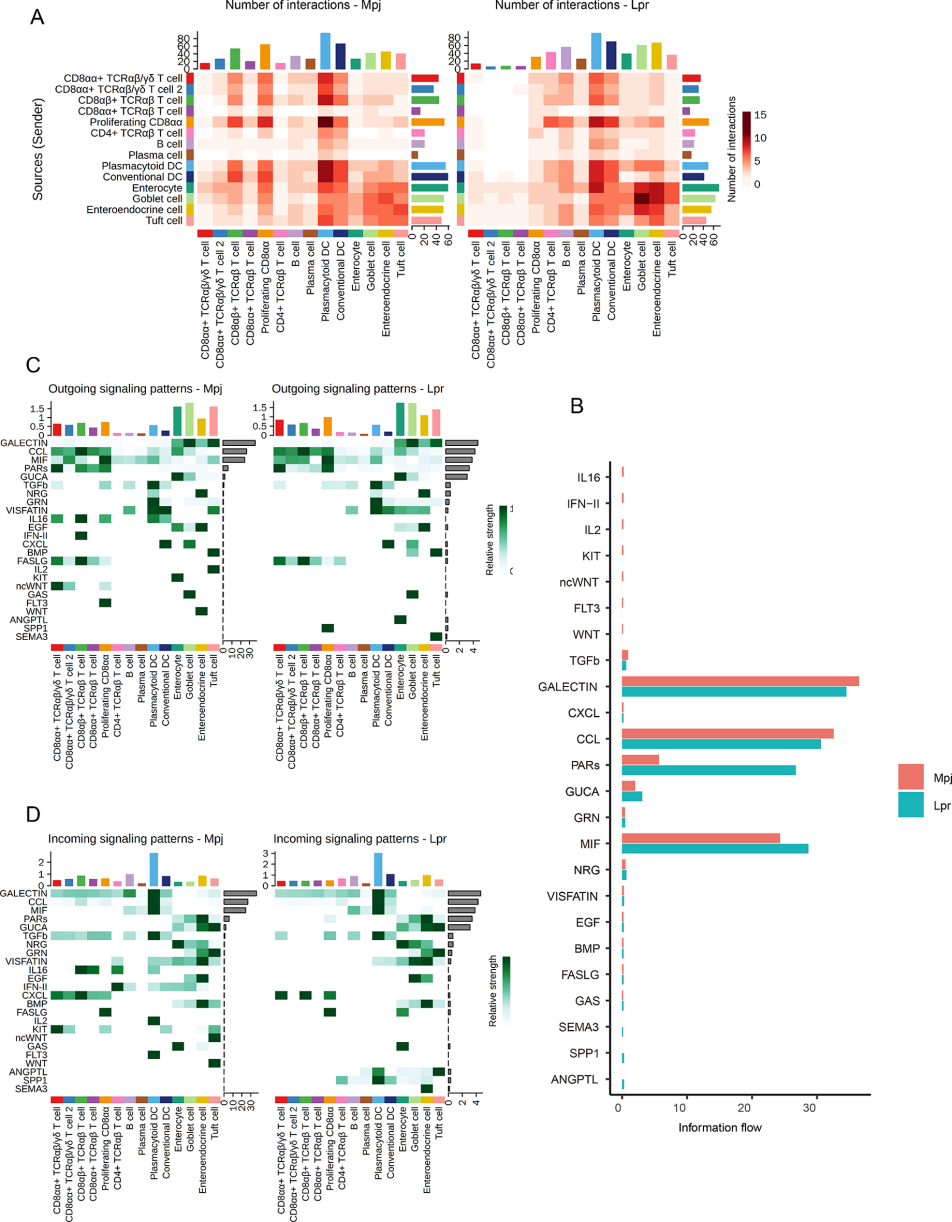
Signaling pathways and information flow were altered in lupus mice intestines. (**A**) Interaction heatmap plotting the total number of interactions in Mpj and Lpr intestinal cell receptor (x axis) and ligand (y axis) interactions. (**B**) Signaling pathways were ranked according to the differences in the overall information flow within the inferred networks between Mpj and Lpr mice intestine. (**C**) Heatmap of outgoing signaling patterns in different clusters. (**D**) Heatmap of incoming signaling patterns across different clusters. The color spectrum in the heatmap of (**C**) and (**D**) indicates the relative strength of signaling pathways within different clusters, which is determined by calculating the network centrality scores using Cellchat to find primary senders (sources) and receivers (targets)

To investigate the crosstalk within the immune and structural cells in Lpr mice intestines. CellChat was used to comprehensively evaluate the interactions between immune and structural cells [24]. The assessment of interaction numbers showed that pDC and cDC cells exhibited a denser interaction network, both in terms of signal sources and reception (Fig. 4A), and a denser interaction network was also observed in Lpr mice structural cells such as enterocytes, goblet cells, and enteroendocrine cells. Almost each CD8 + IELs cluster received a decreased signal crosstalk from immune and structural cells in Lpr mice intestines. A total of 24 pathway information flows were prominently changed in Lpr mice intestines (Fig. 4B and Supplementary Tables 18, 19). Protease-activated receptors (PARs) pathway exhibited the most significant increase among these pathways in Lpr mice. In terms of outgoing signaling patterns, Lpr mice intestines exhibited enhanced signaling strengths in PARs, guanylate cyclase activator (GUCA), angiopoietin-like proteins (ANGPTL), phosphoprotein 1 (SPP1), and semaphorin 3 (SEMA3) pathways based on CellChat, among which ANGPTL, SPP1, and SEMA3 signaling pathways primarily derived from enterocytes, proliferating CD8αα cells and tuft cells, respectively (Fig. 4C). By comparison, decreased signaling strengths in IL16, IL2, KIT, and WNT signaling pathways were identified. DC and structural cells were the main outgoing sources of these signaling pathways. The incoming signaling pathways had similar changes to outgoing signaling patterns (Fig. 4D).

**Fig. 5:**
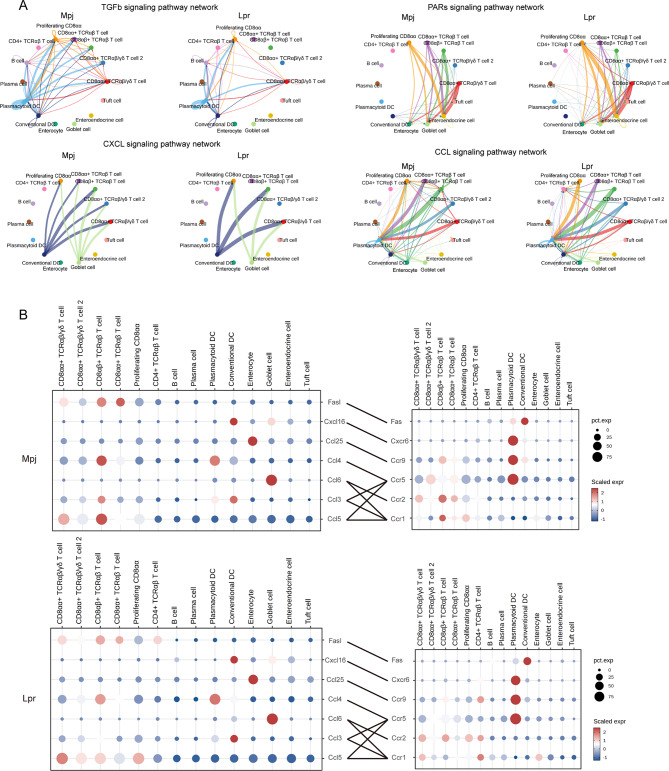
Significantly modified signaling pathways and ligand-receptor analysis reveal the restructuring of the intestinal interactome network in lupus mice. (**A**) Circle plot of the TGFb, PARs, CXCL, and CCL signaling pathways from Cellchat. Receptor-ligand edges were labeled based on communication probability. TGFb, transforming growth factor β; PARs, protease-activated receptors; CXCL, C-X-C motif ligand; CCL, CC chemokine ligand. (**B**) Dot plots showing the expression of ligands (left) and receptors (right) in intestinal cells of Mpj and Lpr mice, with dominant exhibition on chemokines. Color saturation denotes the strength of gene expression on average, while the size of dots corresponds to the proportion of each cell cluster expressing the gene

Further analysis of altered signaling pathways suggested that transforming growth factor β (TGFb) signaling pathway network was prominently compromised in Lpr mice intestines (Fig. 5A). The signaling was mostly derived from pDC and cDC, and primarily targeted CD8 + T cells. Previous studies have indicated the regulatory role of TGFb in the development of CD8αα + TCRαβ IELs [25]. Therefore, the reduced frequency of CD8αα + TCRαβ IELs in the intestines of Lpr mice might be partially attributed to the compromised TGFb signaling. PARs signaling pathway network was significantly enhanced in Lpr mice. Interestingly, we observed a transition in the source of the pathway from cDC in Mpj mice to pDC in Lpr mice. The persistent production of Type I interferon in SLE patients was mainly attributed to the activation of pDCs [26]. The enhanced PARs signaling pathway suggested that the activation of intestinal pDC might also be involved in the pathogenesis of lupus. In addition to pDC, the enhanced PARs signaling pathway was also observed in plasma cells and CD4 + TCRαβ T cells. cDC and goblet cells were the main sources for the C-X-C motif ligand (CXCL) signaling pathway network, the pathway targeted to CD8αα + TCRαβ T cell and CD8αα + TCRαβ/γδ T cell 2 was compromised in Lpr mice. cDC and goblet cells were the main producers of *Cxcl16*, and its receptor *Cxcr6* was primarily expressed in pDC (Fig. 5B). The CC chemokine ligand (CCL) signaling pathway network was also significantly altered in Lpr mice (Fig. 5A, B). The CCL25-CCR9 axis plays a crucial role in the retention of immune cells in intestinal tissues [27]. The enterocyte was the main producer of *Ccl25*, and its receptor *Ccr9* was expressed in most IELs, pDC, and cDC (Fig. 5B). The compromised *Ccl25-Ccr9* axis was observed in CD8αα + TCRαβ T cell and cDC in Lpr mice, while the axis was enhanced for CD4 + TCRαβ T cell. *Ccl6* signals sent out by goblet cells could affect CD8 + IELs or CD4 + IELs via its receptors *Ccr1* and *Ccr2*. The *Ccl6-Ccr2* axis was compromised in CD8αα + TCRαβ T cells while enhanced for proliferating CD8αα and CD4 + TCRαβ T cells in Lpr mice. An increased expression of *Ccr1* was observed for CD8αα + TCRαβ/γδ T cell, CD4 + TCRαβ T cell, and enterocyte in Lpr mice, while the expression was decreased for CD8αβ + TCRαβ T cell, CD8αα + TCRαβ T cell, and proliferating CD8αα. *Ccl6* shared its receptor *Ccr1* with the *Ccl3* primarily sent out by cDC and *Ccl5* primarily sent out by CD8 + IELs.

Together, these findings suggested substantial alterations in communications within the intestinal epithelial immune and structural cells in Lpr mice, implicating the disruption of the gut immune microenvironment in a lupus autoimmune background.

### Significantly decreased epithelial γδT cells in the intestine of lupus mice

**Fig. 6:**
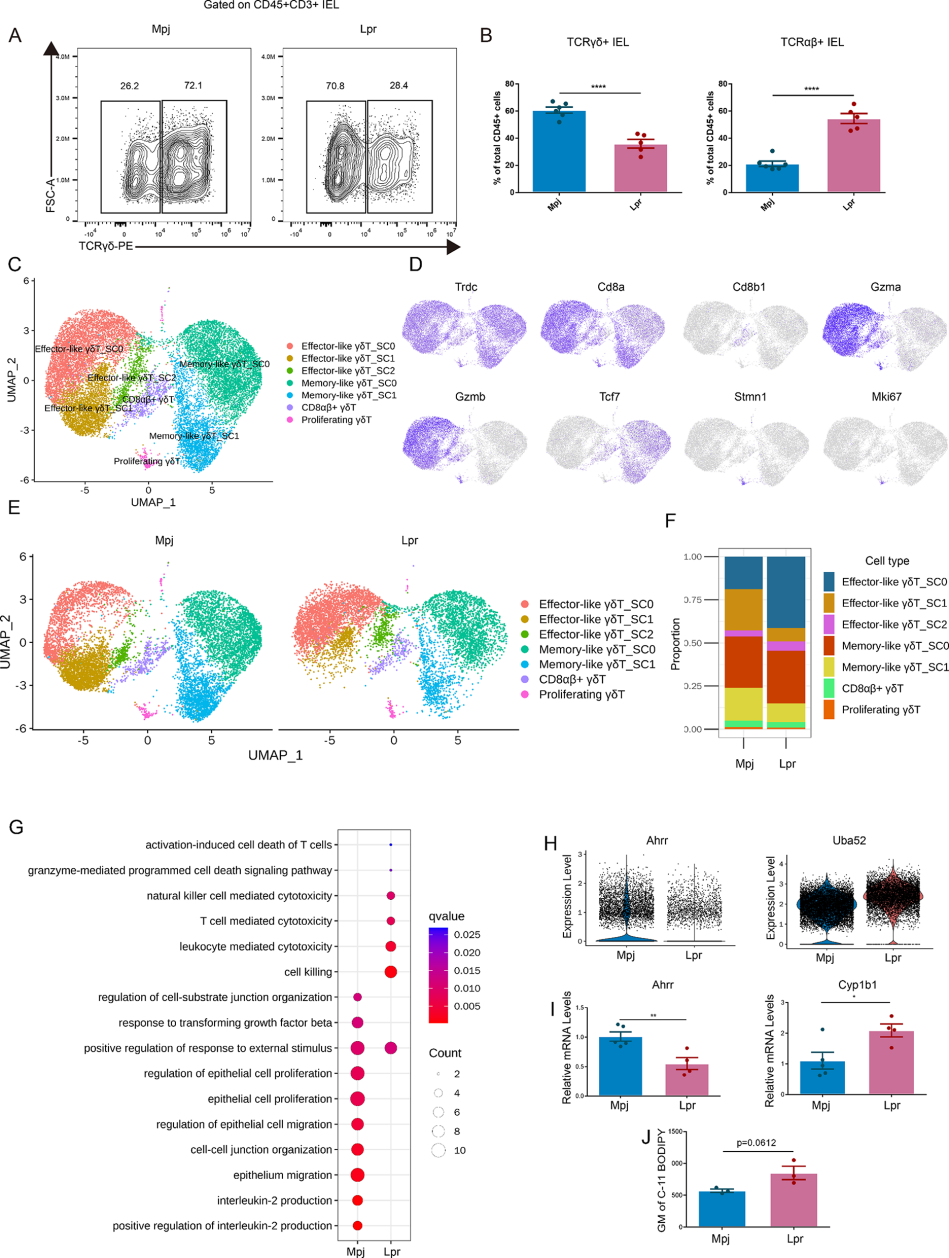
Significantly decreased epithelial γδT in the intestine of lupus. (**A** and **B**) Flow cytometry analyses were conducted in a supplementary group of six Mpj mice and five Lpr mice to examine γδT cell populations. Each dot denotes an individual mouse. (**C**) UMAP plot of 17,259 γδT cells in intestinal epithelial layer. (**D**) Feature plots representing the expression of *Trdc*,* Cd8a*,* Cd8b1*,* Gzma*,* Gzmb*,* Tcf7*, *Stmn1*, and *Mki67*. (**E** and **F**) UMAP plot of γδT subsets (**E**) and frequency of γδT subsets out of total γδT cells (**F**) in Mpj and Lpr mice. (**G**) Bubble diagrams display Gene Ontology (GO) biological process (BP) terms that are enriched in total γδT cells of Mpj and Lpr mice. (**H**) Violin plot illustrating the expression of *Ahrr* and *Uba52* in total γδT cells from Mpj and Lpr mice. Each dot represents one cell. (**I**) *Ahrr* and *Cyp1b1* expression in ileal tissues of Mpj and Lpr mice analyzed by qPCR. (**J**) Quantification of C11 BODIPY staining in γδT cells from Mpj and Lpr mice. Statistical significance in (**B**, **H**-**J**) was determined by student’s *t*-test. Each dot denotes an individual sample. *P < 0.05, **P < 0.01, ***P < 0.001, ****P < 0.0001

The decrease in CD8αα + T cells have been identified in Lpr mice (Fig. 2B, C). Most intestinal CD8αα + T cells are composed of intestinal γδT [8]. scRNA-seq revealed the transcriptional similarities between intestinal γδT and TCRαβ T cells, despite their distinct TCR gene expression. To examine the change of γδT between Mpj and Lpr mice, Flow cytometry analyses were conducted in a supplementary group of six Mpj mice and five Lpr mice, and found that γδT cells were significantly decreased in the intestinal epithelium of Lpr mice, whereas TCRαβ T cells showed a notable increase (Fig. 6A, B). To further investigate the reason causing γδT loss and cellular heterogeneity within γδT in Lpr mice, a total of 17,259 γδT cells were further analyzed (Fig. 6C). UMAP visualization unveiled seven distinct clusters. The identification of γδT was based on the expression of *Trdc*, the widespread expression of *Cd8a* and the limited expression of *Cd8b1* indicating that the majority of γδ T cells are CD8αα + T cells (Fig. 6D). Cytotoxicity-associated gene *Gzma* and *Gzmb* expression within a subset of γδT cells suggested their classification as effector-like γδT cells, and these cells were further divided into three clusters based DEGs. Memory-associated gene *Tcf7* within a subset of γδT cells suggested their classification as memory-like γδT cells, and these cells were further divided into two clusters based DEGs. The expression of *Mki67* and *Stmn1* were used to define proliferating γδT. The total number of γδT cells was decreased in Lpr mice (Fig. 6E), with this reduction primarily attributed to effector-like γδT_SC1 and memory-like γδT_SC1 (Fig. 6F). The DEGs of each γδT cluster were provided in Supplemental Table 20.

Moreover, GO BP analysis revealed that total γδT cells in Lpr mice enriched the terms associated with effector function such as natural killer mediated cytotoxicity, T cell-mediated cytotoxicity, leukocyte mediated cytotoxicity, and cell killing, suggesting an enhanced effector of total γδT in Lpr mice (Fig. 6G and Supplementary Table 21). The pathways associated with cell death including activation-induced cell death of T cells and granzyme-mediated programmed cell death signaling pathway were also enriched in γδT of Lpr mice. Compared to Mpj mice, the pathways significantly down-regulated in the Lpr mice included positive regulation of interleukin-2, regulation of cell-substrate junction organization, epithelial cell proliferation, regulation of epithelial cell migration, and cell-cell junction organization. γδT plays a crucial role in preserving the integrity of the gut epithelium by enhancing the expression of intercellular tight junction proteins such as Zo-1 [28]. Therefore, the impaired gut barrier in lupus might be associated with significantly reduced γδT.

To further investigate whether the γδT loss in Lpr mice could be attributed to the disruption of IELs development and migration. We first examined thymic IEL progenitors. The origin and development of intestinal γδT cells have been controversial. Although some studies have shown that γδT could develop extrathymically [29], most IELs developed from CD4-CD8- thymocytes [8, 30, 31]. Flow cytometry analyses indicated that Mpj and Lpr mice had analogical distributions of CD4-CD8- thymocytes (Supplementary Fig. 9A). In addition, the markers associated with gut homing and tissue retention of γδT, such as Cd103, Ccr9, and Cd69, did not indicate significant differences between Mpj and Lpr mice (Supplementary Fig. 9B), suggesting the γδT loss in Lpr mice is not due to IELs thymic developmental abnormalities or gut homing defects.

DEGs analysis showed that the expression of aryl-hydrocarbon receptor repressor (*Ahrr*) was prominently down-regulated in total γδT of Lpr mice (Fig. 6H). We confirmed the decreased expression of *Ahrr* in the ileal tissues of Lpr mice by qPCR (Fig. 6I). A recent study showed that *Ahrr* deficiency could lead to an overproduction of reactive oxygen species (ROS), and cause oxidative stress and ferroptosis of intestinal epithelial γδT [32]. One of the primary effector molecules induced by aryl-hydrocarbon receptor (*AHR*) is cytochrome P450 family 1 member B1 (*CYP1B1*), belonging to the cytochrome P450 family of monooxygenases, which is considered as an oxidative stress marker [33, 34]. The reduced expression of *Ahrr* implies an increased activity of *Ahr*, which is supported by the high expression of *Cyp1b1* in the ileal tissues of Lpr mice (Fig. 6I). Further analysis also indicated the high *Uba52*, an oxidative stress-inducing ubiquitin gene [35], in total γδT of Lpr mice. To further test the oxidative stress and ferroptosis of intestinal γδT cells in the epithelium. We stained intestinal γδT of Mpj and Lpr mice with C11 BODIPY staining, a marker of lipid peroxidation and ferroptosis [36]. Intestinal γδT cells from Lpr mice showed a marked increase lipid peroxidation and ferroptosis compared with the same cells from Mpj mice (Fig. 6J).

Taken together, these data indicated a significant decrease of intestinal epithelial γδT in Lpr mice, which might be one of the reasons causing compromised integrity of the gut barrier in lupus. In addition, the decreased γδT might be attributed to the oxidative stress caused by the reduced expression of AHRR.

### Supplementing the diet with selenium improved the gut barrier and lupus symptoms via mitigating the loss of intestinal epithelial γδT cells

**Fig. 7:**
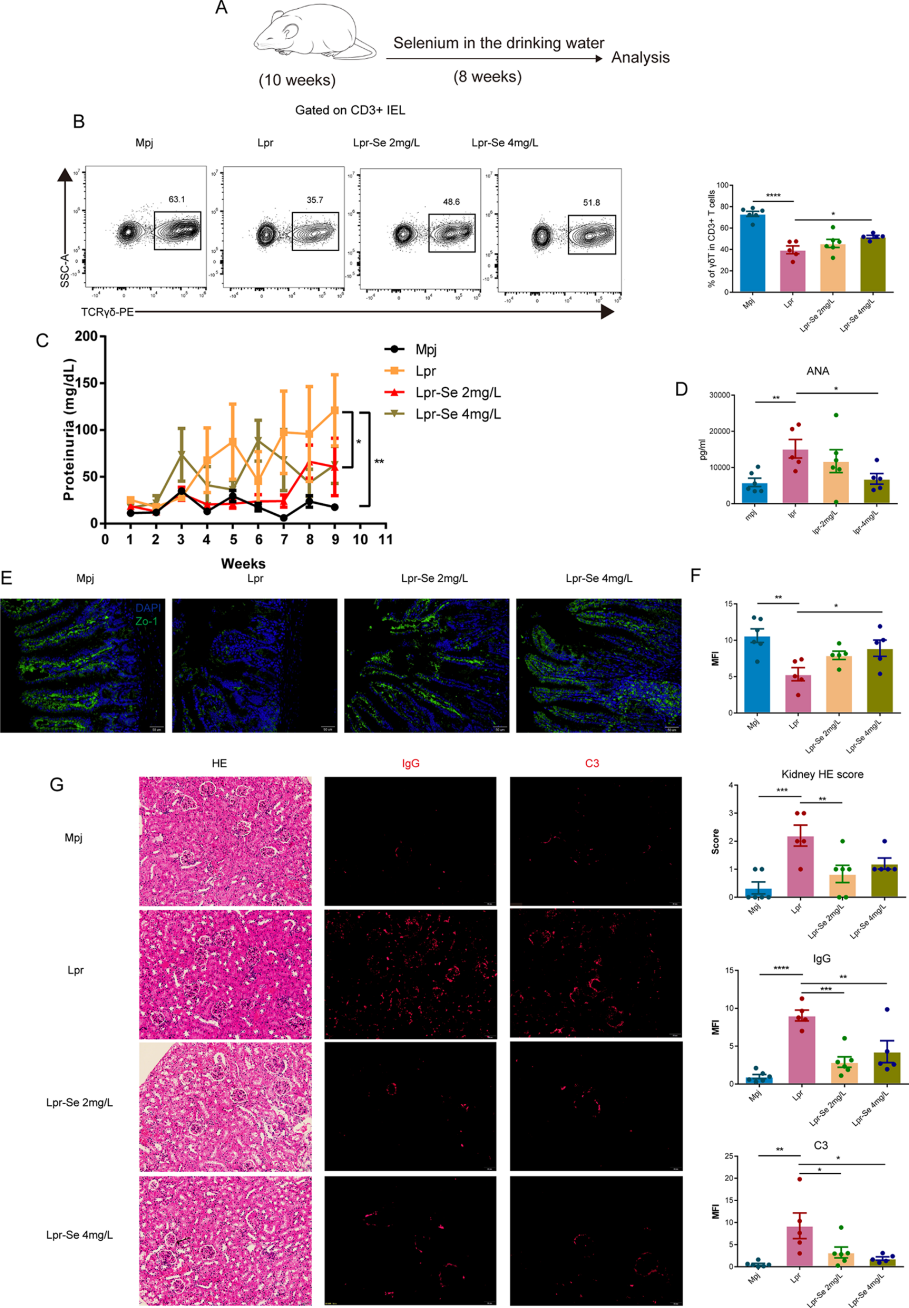
Dietary selenium supplementation rescues γδT loss and ameliorates lupus symptoms in Lpr mice. (**A**) Lpr mice were administered drinking water supplemented with selenium at concentrations of 2 mg/L and 4 mg/L for 8 weeks, respectively. Se, selenium. (**B**) Representative flow cytometry plots and quantification of γδT in CD3 + T cells from Mpj and Lpr mice with and without dietary selenium supplementation. (**C** and **D**) Proteinuria (**C**) and ANA (**D**) from Mpj and Lpr mice with and without dietary selenium supplementation. ANA, anti-nuclear antibodies. (**E** and **F**) Representative plot and quantification of Zo-1 expression based on immunohistochemical staining. MFI, Mean Fluorescence Intensity; Zo-1, zona occludens-1. (**G**) Representative plot and quantification of H&E staining, IgG and C3 deposition in the kidneys. Statistical significance in (**B**-**D**, **F**-**G**) was determined by one-way ANOVA with dunnett post hoc test. Each dot denotes an individual mouse. *P < 0.05, **P < 0.01, ***P < 0.001, ****P < 0.0001

Selenium performs diverse cellular functions, encompassing the regulation of ROS production and the maintenance of redox homeostasis and, thereby constraining lipid peroxidation [37]. To further examine whether γδT loss in Lpr mice was due to oxidative stress, we tested whether supplementing the diet with selenium could mitigate the loss of γδT. Ten-week-old Lpr mice were administered drinking water supplemented with selenium at concentrations of 2 and 4 mg/L for 8 weeks, respectively (Fig. 7A). We found that selenium supplementation prominently increased the frequency of γδT in Lpr mice compared to those consuming ordinary drinking water (Fig. 7B). Selenium supplementation also augmented the expression of Zo-1 based on immunohistochemical staining, suggesting an improved integrity of gut barrier in Lpr mice (Fig. 7E, F). In addition, treatment with selenium resulted in a decrease in kidney H&E scores, proteinuria, and the concentration of autoantibody ANA in serum. Reduced IgG and C3 deposits in the kidneys were also observed in Lpr mice receiving selenium supplementation compared to those consuming ordinary drinking water (Fig. 7G). These results suggested that the γδT loss in the intestinal epithelium of Lpr mice could be attributed to oxidative stress. Antioxidant treatment with selenium not only mitigates the loss of γδT, improves the gut barrier and ameliorates the lupus symptoms in Lpr mice.

### IFN-γ contributed to the reduced expression of *AHRR*

**Fig. 8:**
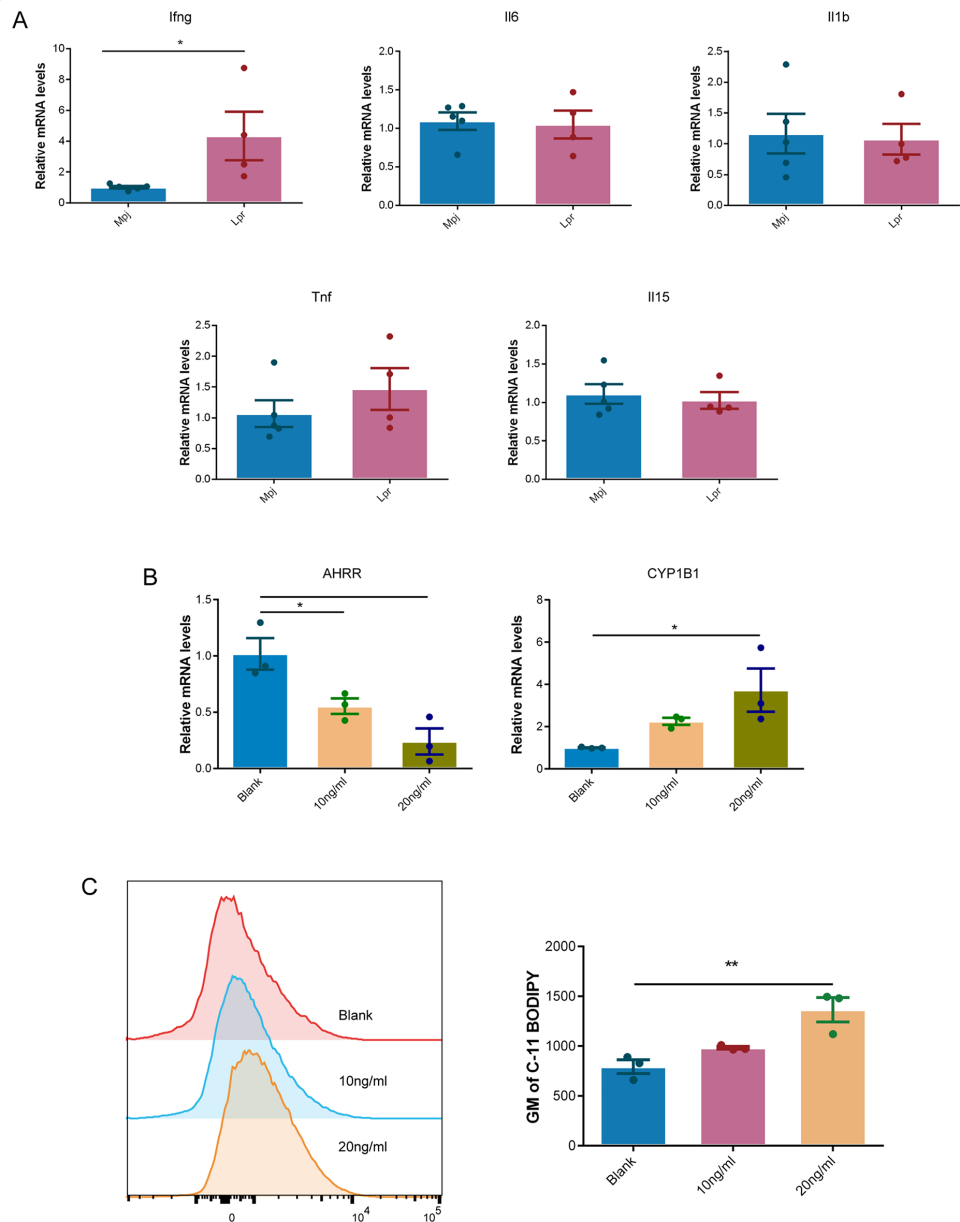
IFN-γ contributed to the reduced expression of AHRR. (**A**) Inflammatory cytokines expression in ileal tissues of Mpj and Lpr mice analyzed by qPCR. (**B**) *AHRR* and *CYP1B1* expression in Jurakt cells induced by different concentrations of IFN-γ. *AHRR*, Aryl Hydrocarbon Receptor Repressor. (**C**) Representative flow cytometry result and quantification of C11 BODIPY staining in Jurkat cells induced by different concentrations of IFN-γ. Statistical significance in (**A**) was determined by student’s *t*-test and (**B** and **C**) was determined by a one-way ANOVA test with Dunnett’s multiple comparisons test. Each dot denotes an individual sample. *P < 0.05, **P < 0.01, ***P < 0.001, ****P < 0.0001

Various inflammatory cytokines have been shown to lead to oxidative stress [38]. To investigate whether γδT oxidative stress induced by the reduced expression of AHRR was associated with augmented inflammatory cytokines, we tested multiple inflammatory cytokines expression in the ileum tissues of Lpr mice and found that *Ifng* exhibited the most notable increase in Lpr mice compared to Mpj mice (Fig. 8A). Subsequently, we directly stimulated Jurkat cells with different concentrations of interferon-γ (IFN-γ) and assessed the expression of *AHRR*. We observed a significant downregulation of *AHRR* in Jurkat cells induced by IFN-γ with a dose-dependent effect (Fig. 8B). Furthermore, the expression of *CYP1B1* significantly increased following IFN-γ stimulation, indicating an overactivation of *AHR*. C11 BODIPY staining showed an increased lipid peroxidation and ferroptosis in Jurkat cells induced by IFN-γ (Fig. 8C). These data indicated that IFN-γ stimulation might contribute to the reduced expression of *Ahrr* in γδT cells from intestinal epithelial of Lpr mice.

## Discussion

In this study, we conducted scRNA-seq analyses of immune cells and structural cells in the intestinal epithelium of Lpr mice, systematically elucidating the compositional and functional alterations of these cells in the lupus autoimmune background, as well as the disrupted intercellular crosstalk patterns compared to control mice. Additionally, we investigated the disruption of the epithelial lymphocytes as a contributing factor to the compromise of the gut barrier in lupus.

Our study showed a significant alteration in the composition of CD8 + IELs, manifested by a shift in proportion from CD8αα + IELs in Mpj mice to CD8αβ + IELs in Lpr mice. Interestingly, similar changes in the IELs have been reported in other autoimmune diseases. Crohn’s disease presented a significant decrease in CD8αα + IELs, a finding derived from single-cell sequencing analysis [39]. A recent study indicated that CD8αα + γδT can additionally contribute to the maintenance of Paneth cell viability through the secretion of apoptosis inhibitor 5 (API5) in Crohn’s disease [40]. CD8αα homodimers display a strong binding affinity with the thymus leukemia (TL) Ag, a nonclassical MHC class I molecule [41–43], and play a critical role in maintaining immune tolerance through negatively regulating the activation of T cells and reducing the sensitivity of TCR [44]. The notable reduction of CD8αα + IELs in Lpr mice might implicate the disruption of mucosal immune tolerance. In addition, several CD8 + IEL subsets exhibiting significantly augmented effector function were markedly enriched within the intestinal epithelial layer in Lpr mice. Further studies are needed to investigate the impact of the effector CD8 + IELs on the pathogenesis of lupus.

Intestinal structural cells play a critical role in gut barrier maintenance and the absorption of nutrients [45, 46]. Our data indicated that despite no significant changes in the proportion of enterocyte subgroups classified along the villus axis, there were distinct functional alterations. GO enrichment analyses uncovered significant downregulation of pathways associated with tight junctions between intestinal epithelial cells, suggesting disruption of the gut barrier in lupus mice (Fig. 3E). Previous studies have reported significant disruption of the gut barrier in both SLE patients and SLE models [23, 47–49]. Barrier disruption-induced bacterial translocation plays a critical role in SLE pathogenesis [16]. Additionally, lipid metabolism-related pathways were significantly enriched in the enterocytes of Lpr mice, with module scores indicating a substantial enhancement in lipid absorption function, which might in part contribute to metabolic disturbances in lupus. Furthermore, the significantly enhanced cellular lipid metabolism is susceptible to oxidative stress, as the process may be accompanied by an increase in polyunsaturated fatty acids [50, 51]. Consistently, our study showed that pathways related to oxidative stress were markedly enriched in the enterocytes of Lpr mice, which suggested that oxidative stress might be an important risk in the intestine of Lpr mice.

**Fig. 9:**
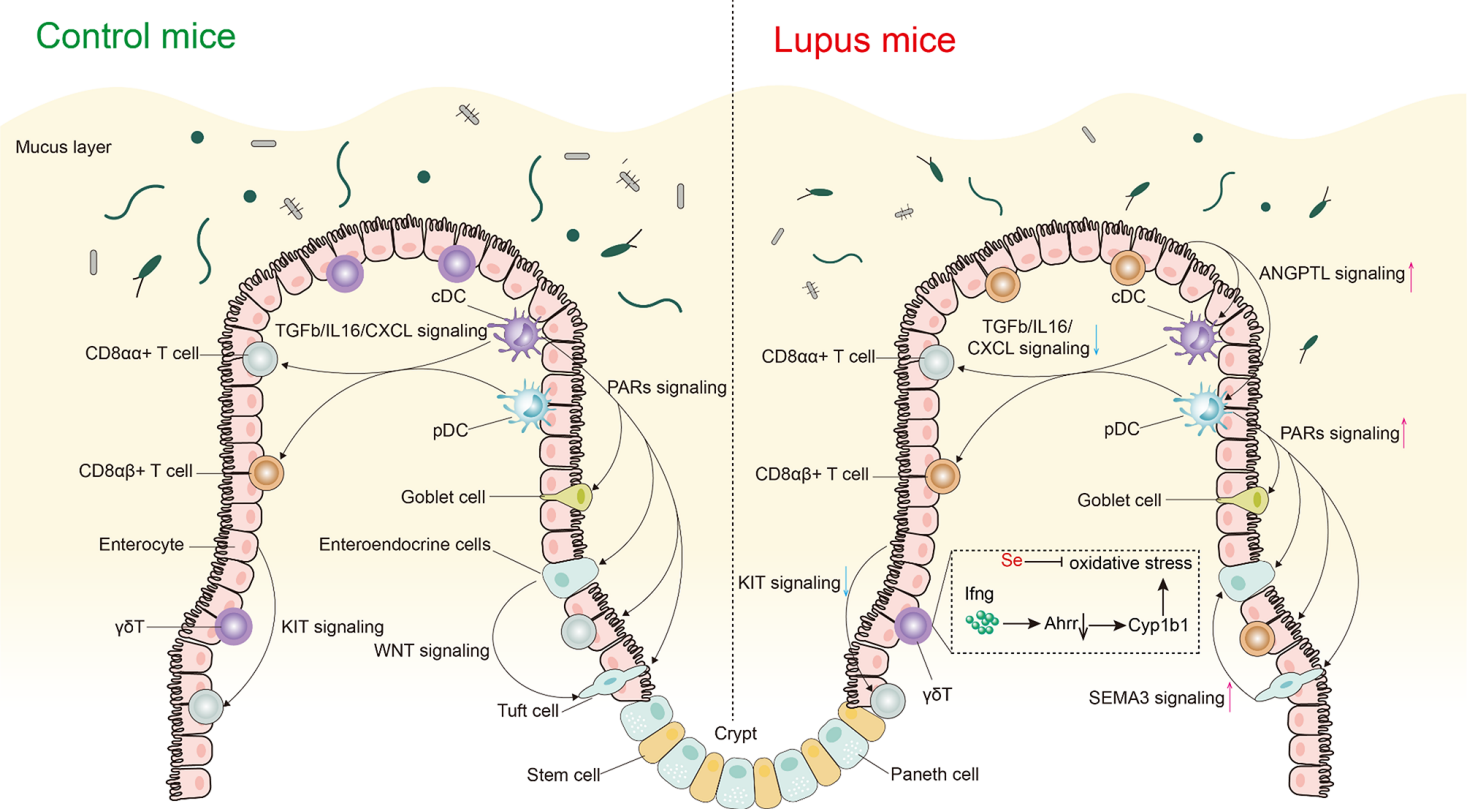
Graphical summary of disrupted immune and structural milieu in the intestinal epithelium of Mpj and Lpr mice. A conspicuous imbalance in CD8 + IEL was observed in Lpr mice, characterized by a notable reduction in CD8αα + IELs and a significant elevation in CD8αβ + IELs. Additionally, intercellular communication patterns based on CellChat between intestinal epithelial immune and structural cells were found to be specifically altered in Lpr mice. pDC and cDC act as the central communication hub, engaging in interactions with IELs and structural cells. It was noted that the TGFb/IL16/CXCL signaling pathway, which is initiated by DCs and affects CD8 + IELs, demonstrated a reduced interaction in Lpr mice. KIT signaling, originating from enterocyte and targeting CD8αα + IEL, exhibited diminishing interactions in Lpr mice. WNT signaling, initiated by enteroendocrine cells and affects tuft cells, also demonstrated reduced interaction in Lpr mice. Augmented signaling in Lpr mice included SEMA3/ANGPTL/PARs signaling. Of note, the main sources of PARs signaling were cDC in Mpj mice and pDC in Lpr mice. Furthermore, a marked reduction in γδT cells was observed in Lpr mice, which was associated with reduced AHRR expression and subsequent oxidative stress and ferroptosis, and increased IFN-γ levels in the intestines of Lpr mice contributed to the reduced expression of AHRR. Antioxidant selenium effectively reversed the loss of γδT in Lpr mice. TGFb, transforming growth factor β; CXCL, C-X-C motif ligand; SEMA3, semaphorin 3; ANGPTL, angiopoietin-like proteins; PARs, protease-activated receptors; Ahrr, Aryl hydrocarbon receptor repressor; DC, dendritic cells; Se, selenium

Intercellular communication analyses using CellChat revealed several markedly altered signaling pathways in Lpr mice (Fig. 9). For instance, we observed a weakening of the TGFb/IL16/CXCL/WNT signaling pathway in Lpr mice. TGFb has been extensively documented as a key cytokine for maintaining intestinal immune homeostasis and was critical for the development of intestinal epithelial immune cells [25, 52]. WNT signaling plays a crucial role in maintaining Lgr5 + intestinal stem-cell self-renewal and gut integrity [53, 54]. The weakening of these pathways implies compromised self-renewal and homeostasis maintenance in lupus intestines. Furthermore, we observed that the PARs signaling pathway was notably enhanced in Lpr mice, suggesting that the intestines of Lpr mice may be exposed to a higher number of proteases. There is a shift in the signaling sources of the PARs pathway, with cDC as the signal source in Mpj mice, and pDC in Lpr mice, where pDC cells are the primary immune cells responsible for type I interferon production in lupus pathogenesis [55]. Our findings indicated that pDCs might also play a key role in the disruption of the intestinal immune microenvironment in lupus.

A notable γδT loss was observed in Lpr mice. A recent study has demonstrated that the knockout of AHRR significantly contributed to intestinal γδT loss leading to pronounced oxidative stress. *Ahrr*-deficient mice are susceptible to *Clostridium difficile* and reduced *AHRR* expression has also been observed in inflammatory bowel disease (IBD) [32]. Our results suggested that the loss of γδT caused by low *AHRR* expression may not be limited to intestinal disorders and may also contribute to extra-intestinal autoimmune diseases such as lupus. Additionally, γδT plays a crucial role in mucosal immune tolerance. Following the clearance of gut microbiota with antibiotics in mice, the development of γδT is significantly impacted, leading to a disruption in mucosal immune tolerance [56]. Thus, we speculate that the loss of γδT in lupus may not only contribute to barrier disruption but also be associated with the dysregulation of mucosal immune tolerance in lupus. Further research is required to confirm this hypothesis.

Our study has certain limitations, including the lack of analysis of immune cells in the intestinal lamina propria. Furthermore, while we have identified IFN-γ as an important factor causing reduced *Ahrr* in lupus intestines, the reasons for the significant increase of IFNG in intestines of lupus still require further investigation.

In summary, our study found a conspicuous imbalance within the CD8 + IELs subpopulation in the intestinal epithelium of lupus mice. Additionally, CD8 + IELs subsets exhibiting significantly augmented effector function were found to be markedly enriched in Lpr mice. Intercellular communication patterns between intestinal epithelial immune and structural cells were found to be specifically altered in lupus mice. Furthermore, the compromised gut barrier of lupus mice might be attributed to intestinal epithelial γδT loss associated with reduced AHRR expression and subsequent oxidative stress. Antioxidants effectively reversed γδT loss in lupus mice, improved the gut barrier, and alleviated lupus symptoms. Our high-resolution single-cell atlas advances the understanding of the lupus intestinal immune microenvironment, and provides new insights into lupus pathogenesis mediated by intestinal immune dysregulation.

## Materials and methods

### Mice

Twenty-week-old female Mpj mice and Lpr mice were obtained from SPF Biotechnology Co., Ltd. For the single-cell analysis, we used three mice per group for both the Mpj and Lpr strains. The number of mice used in the other experiments is specified in detail in the relevant sections of the paper. The study used only female mice. All mice were raised in specific pathogen-free conditions at the animal facilities of the Institute of Dermatology, Chinese Academy of Medical Sciences, and all required sterilization measures were conducted, which included autoclaving cages, bedding, nestlets, as well as food and water. Cervical dislocation was used for mouse euthanasia. All experiments adhered to the animal ethical and care guidelines established by the Institute of Dermatology, Chinese Academy of Medical Sciences (2023-DW-031).

### Flow cytometry plot analysis

Flow cytometry was employed to assess the expression of cell surface markers in immune cells. To identify distinct IELs subpopulations, A single-cell suspension of the small intestinal epithelium was prepared and suspended in PBS as the buffer. IELs were stained with anti-CD45-Percp-Cy5.5 (BD Pharminge, 550994), anti-CD3-eF450 (BD Pharminge, 560801), anti-CD8α-APC-Cy7 (Biolegend, 100714), anti-CD8β-BV510 (Biolegend, 126631), anti-CD4 (BD Pharminge, 557307), anti-TCR γ/δ-PE (Biolegend, 118107), anti-CD103-BV421 (Biolegend, 121421). Flow cytometry data were collected using a CYTEK NL-3000 (CYTEK Biosciences) flow cytometer and analyzed using FlowJo software.

### Selenium supplementation for mice

Lpr mice were provided with water containing Selenium (2 mg/L or 4 mg/L, Sigma, cat: S3132) and were kept on these diets for 8 weeks before the experiments. Mpj and Lpr mice raised on standard water served as controls.

### Assessment of lupus disease severity and gut barrier

To assess the severity of lupus disease in Lpr mice, serum samples were collected via the orbital vein after anesthesia at the end of the experiment. Serum levels of ANA were measured using an ELISA kit from CUSABIO (CSB-E12912m). Quantitative analysis of proteinuria was performed using a Bradford assay kit acquired from Nanjing Jiancheng Bioengineering Institute (C035-2-1). Kidney sections were processed for histopathological analysis using Hematoxylin and Eosin (H&E) staining. Briefly, the kidney tissues were first fixed in 10% formalin, embedded in paraffin, and sectioned at a thickness of 3 μm. The sections were then deparaffinized, rehydrated through graded alcohols, and stained with Hematoxylin for 5 min, followed by Eosin for 15s. After staining, the sections were dehydrated, cleared in xylene, and mounted with a coverslip. The stained sections were examined under a light microscope to assess renal histopathology. The glomerulonephritis pathological scores were determined as the mean of ten randomly selected glomeruli results. Briefly, a scoring system was employed [57, 58], with 0 representing normal; 1 indicating cell proliferation or infiltration; 2 indicating membrane proliferation, hyaline deposition, or lobulation, and 3 indicating global hyalinosis or crescent formation. Furthermore, immune complex depositions, including IgG (HRP-conjugated anti-mouse IgG, 1:2000, Abcam) and C3 (rabbit anti-C3 antibody, 1:5000, Abcam), in the kidney sections embedded in paraffin were evaluated through immunofluorescence, a brief summary of the immunofluorescence methodology is provided as follows [59]: paraffin tissue sections underwent antigen retrieval followed by treatment with primary and secondary antibodies and staining using an Opal 7-color IHC detection kit (NEL811001KT, PerkinElmer, Hopkinton, MA). Immunofluorescence was also used to assess the expression of junction protein ZO-1(1: 200, ab276131, Abcam), a marker associated with the intestinal barrier.

### Real-time qPCR

RNA was isolated from ileum or Jurkat cells using the RNAiso Plus.

(9109, TaKaRa) following the manufacturer’s guidelines. The RNA concentration and quality were assessed using a NanoDrop spectrophotometer (ND-2000, Thermo Fisher Scientific). cDNA was synthesized from 1 µg of RNA using HiScript III RT SuperMix for qPCR (Vazyme, R323-01) following the provided protocol. For qPCR analysis, ChamQ Universal SYBR qPCR Master Mix (Vazyme, Q711-02) was used. The expression of various genes was analyzed using the LightCycler 96 (Roche) thermocycler. The calculation of the relative expression of target genes was calculated by 2^^−ΔΔCt^, where ΔΔCt represents the difference between the ΔCt values of the experimental and control groups, with *Actb* as the internal reference gene for normalization. The following genes were analyzed in both human and mouse samples: *CYP1B1* and *AHRR*. The remaining genes, including *Ahrr*,* Il15*,* Kgf*,* Ifng*,* Il6*,* Il1b*,* Tnf*,* Cyp1b1*,and *Zo-1* were specifically analyzed in mouse samples. A comprehensive listing of primer sequences used in this investigation can be found in Supplementary Table 22.

### Stimulation of Jurkat cells with IFN-γ

Jurkat cells were cultured in RPMI-1640 medium supplemented with 10% fetal bovine serum (FBS) and 1% penicillin-streptomycin. The cells were incubated with 10 ng/mL or 20 ng/mL IFN-γ, or left untreated (blank group) for 3 days at 37 °C in a 5% CO₂ incubator. Following the stimulation, the expression levels of AHRR and CYP1B1 were evaluated by qPCR, and lipid peroxidation as well as ferroptosis were analyzed using C11 BODIPY staining.

### C11 BODIPY staining

We surface-stained IELs from Mpj and Lpr mice to distinguish γδT cells. These IELs or Jurkat cells were subjected to a 30-minute incubation at 37 °C with C11 BODIPY (Thermo Fisher), followed by two PBS washes before analysis via flow cytometry.

### Isolation of intraepithelial immune cells and structural cells

Intraepithelial immune cells and structural cells were isolated from small intestinal epithelium. The cell isolation procedure followed methods outlined in previous literature [19, 32]. In brief, after euthanasia, the ileum to jejunum from the small intestine was extracted from Mpj and Lpr mice. They were rinsed in Hanks’ Balanced Salt Solution (HBSS) solution to remove feces. Peyer’s patches were removed, and then the small intestine tissue was longitudinally cut into pieces of approximately 1 cm. The tissue was subsequently digested in a digestion solution (RPMI 1640 culture media with 10% FBS, 2 mM ethylenediaminetetraacetic acid (EDTA)) and incubated for 30 min at 37 °C with 200 rpm rotation in an incubator. The flow through was subjected to a 100-µm cell strainer and 40-80% Percoll gradient centrifugation for increasing the proportion of IELs.

### Single-cell RNA sequencing

Sequencing analysis of immune cells and structural cells sourced from the intestinal epithelial layer was performed using the 10x Genomics platform. Using the Cell Ranger pipeline, Single-cell RNA sequencing data was processed to align reads and produce gene-cell expression matrices (https://support.10xgenomics.com/single-cell-gene-expression/software/overview/welcome). The R software package Seurat was used to perform data analyses in the study (http://satijalab.org/seurat/) [60]. Low-quality cells from the data were first excluded if the genes detected were fewer than 200 or greater than 5,000. Cells were then filtered to have at most 20% mitochondrial gene expression and at most 10% red cell gene expression. A total of 38,737 cells were acquired. In addition, genes not detected in a minimum of three single cells were omitted from further analysis. Normalization and scaling of the filtered gene expression matrix were conducted using a scaling factor of 10,000. Subsequently, the FindVariableFeatures function was employed to generate variable genes. Moreover, the initial 3,000 highly variable genes were chosen for principal component analysis (PCA). The harmony package was used to remove the batch effect among distinct samples (https://github.com/immunogenomics/harmony) [61]. The ElbowPlot function was used to identify meaningful principal components (PCs), and the first 50 PCs were used to perform UMAP (uniform manifold approximation and projection) clustering analysis to reduce the dimensions to two. Next, the same 50 PCs were used to construct a shared nearest-neighbor graph (SNN; Find Neighbors), and this SNN was employed to cluster the dataset (Find Clusters, resolution = 0.5). The marker genes in each cluster were determined using the FindAllMarkers function, which compared one cluster with other all cells. Cell types in the intestinal epithelium were annotated accorded to the cluster markers. The average gene expression of each cluster was determined using the function AverageExpression. Stacked violin plots feature plots were utilized to visualize the expression levels of certain genes. The sub-clustering analyses were performed by following the same steps as above. In UMAP, γδT did not exhibit clear differentiation from TCRαβ T cells due to their high transcriptional similarities. To proceed with subsequent analysis on all γδT, those expressing the *Trdc* gene were specifically chosen as the subset for further investigation. Furthermore, the clusterProfiler package was employed to conduct Gene Ontology (GO) functional analysis as previously described, focusing on biological process [62]. Monocle 2 was used to perform pseudotime trajectory analysis to infer potential differentiation associations between cell subtypes [63].

### Intercellular communication analysis

Intercellular communication analysis within intestinal epithelial immune and structural cells was conducted through CellChat (v.1.6.1) based on the analysis of ligand-receptor interactions [24]. The normalized Seurat data were used as the CellChat object. The computeCommunProb function was utilized to calculate communication probabilities, illustrating cell interactions in terms of both the weight and number of interactions. A graphical representation of the interaction frequency between each pair of cell types was generated utilizing heatmap and circle interactome visualizations based on the sources and receptors of the interactions. Additionally, expression patterns across all cell types for selected ligand-receptor pairs were illustrated in a dot plot.

### Statistical analysis

The graphical representation and statistical analysis of data were performed through GraphPad Prism 6 (GraphPad Software). Mean values accompanied by the standard error of the mean (SEM) were presented. Group comparisons were executed using a two-tailed Student’s t-test. Furthermore, multiple comparisons were conducted through a two-way ANOVA followed by Dunnett’s multiple comparison test. Statistical significance was established at *p* < 0.05, indicated by *, **, ***, and **** for p-values less than 0.05, 0.01, 0.001, and 0.0001 respectively, whereas “ns” represented nonsignificant differences.

## Electronic supplementary material

Below is the link to the electronic supplementary material.

Supplementary Material 1

Supplementary Material 2

## Data Availability

The authors assert that upon request, all pertinent data and code necessary for data analysis will be provided.
