# Supplementary material for: Single-cell analyses of intestinal epithelium reveal the dysregulation of gut immune microenvironment in systemic lupus erythematosus
Source: J Transl Med. 2025 Jan 27;23:118. doi: 10.1186/s12967-025-06147-5 (PMC11773722; doi:10.1186/s12967-025-06147-5)
Supplement: Supplementary file 1 — Supplementary Material 1 [file 12967_2025_6147_MOESM1_ESM.pdf]

Supplemental figures

Single-cell analyses of intestinal epithelium reveal the dysregulation of gut immune microenvironment in systemic lupus erythematosus

Qiaolin Wang [1,2], Yutong Wu [1,3], Lianlian Ouyang [3], Xiaoli Min [1,2], Meiling Zheng [1,2], Xiaoyun Chen [1,2], Zhi Hu [1,2], Shuang Yang [3], Wenjuan Jiang [1,2], Lingyu Gao [1,2], Sujie Jia [4], Qianjin Lu [1,2,3*], Ming Zhao [1,2,3*]

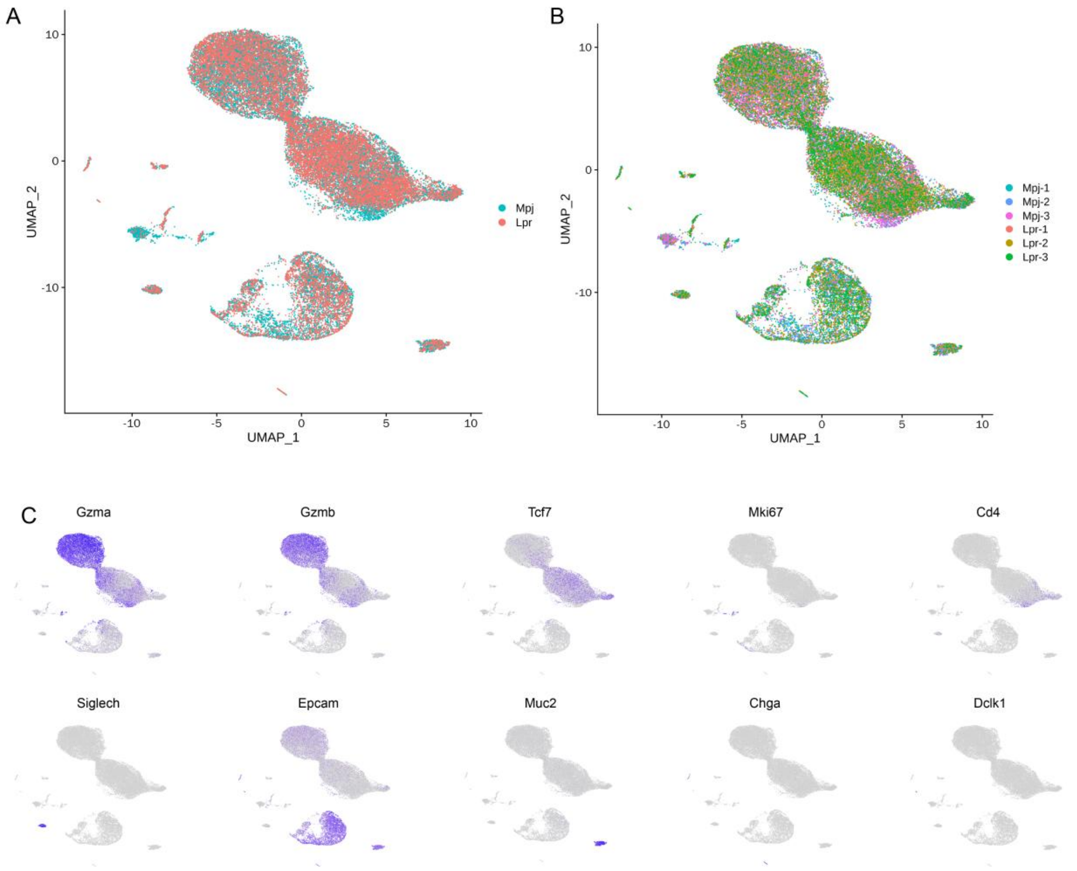

Supplementary Figure 1. scRNA-seq reveals the distribution of cells across various groups and individual samples. (A and B) UMAP plots shows the distribution of immune cell and epithelial structural cell across various groups (A) and individual samples (B). (C) Feature plots representing expression of *Gzma, Gzmb, Tcf7, Mki67, Cd4, Siglech, Epcam, Muc2, Chga, Dclk1*.

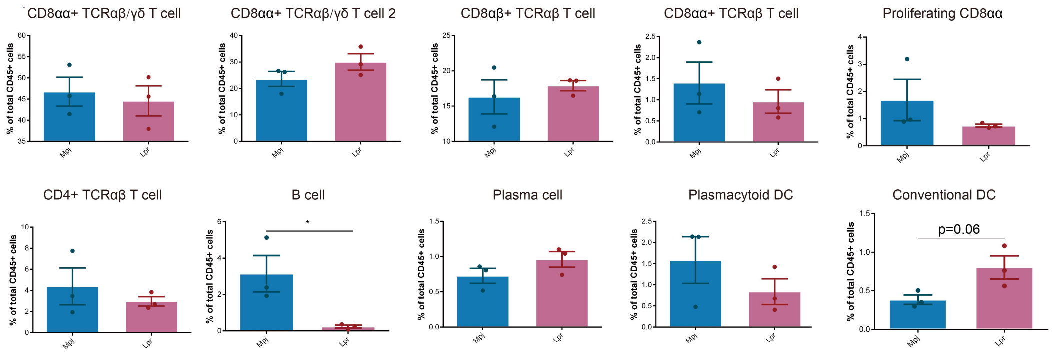

Supplementary Figure 2. scRNA-seq reveals altered immune cell proportion. Histogram plots illustrating the comparison of major immune cell types between Mpj (n=3) and Lpr (n=3) based on scRNA-seq. The histograms for Mpj are presented in blue, histograms for Lpr are in red. Data were presented as mean ± SEM. *P < 0.05, **P < 0.01, ***P < 0.001, and ****P < 0.0001 by Student's t test.

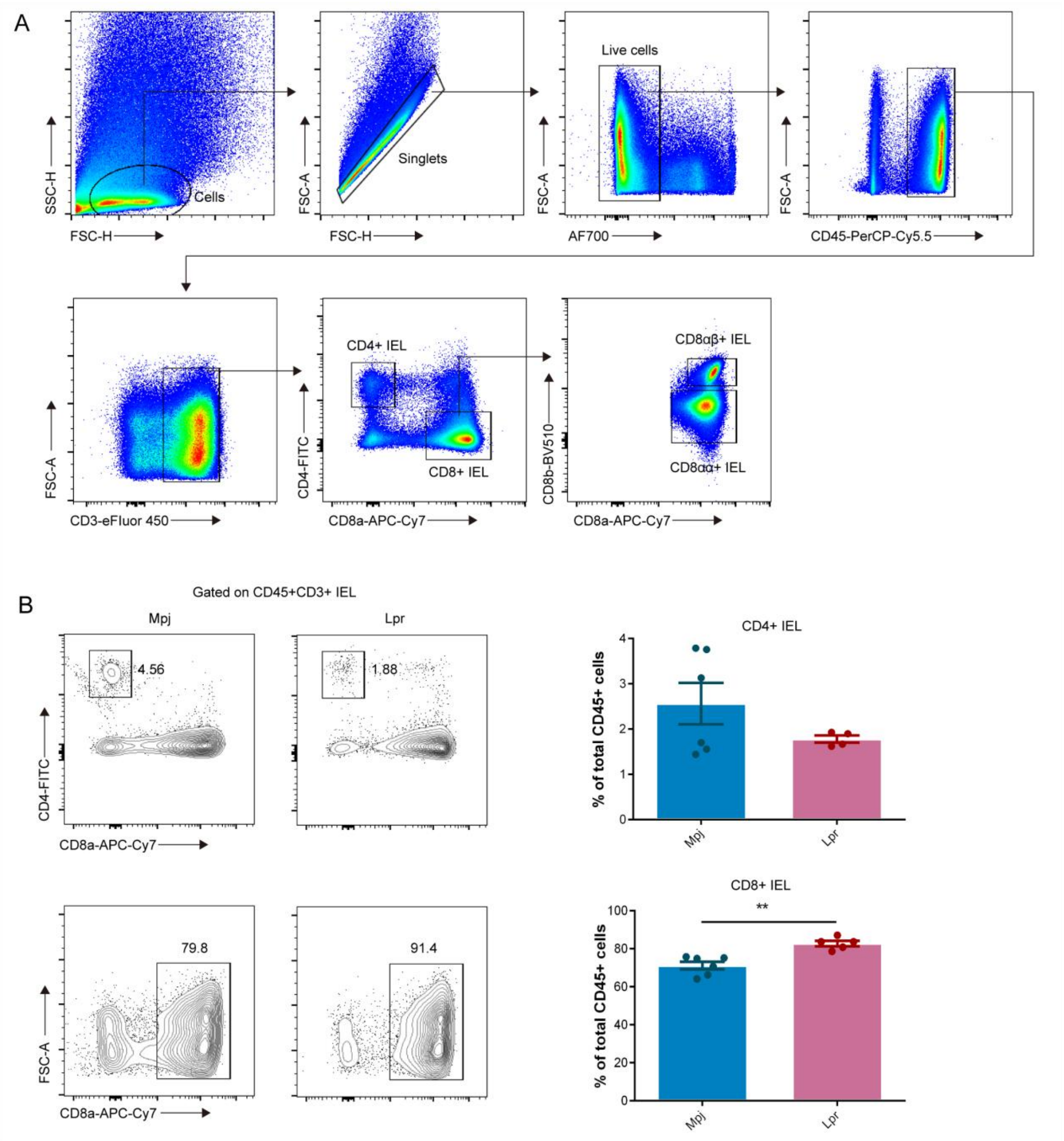

Supplementary Figure 3. Gating strategy of intestinal intraepithelial lymphocytes and the comparison oT cells. (A) Representative flow cytometry analysis gating scheme for intestinal intraepithelial immune cells. (B) Flow cytometry analyses of CD4+ and CD8+ IEL between Mpj and Lpr (Gated on CD45+CD3+ IEL). Each point represents an individual sample. Data were presented as mean ± SEM. *P < 0.05, **P < 0.01, ***P < 0.001, and ****P < 0.0001 by Student's t test.

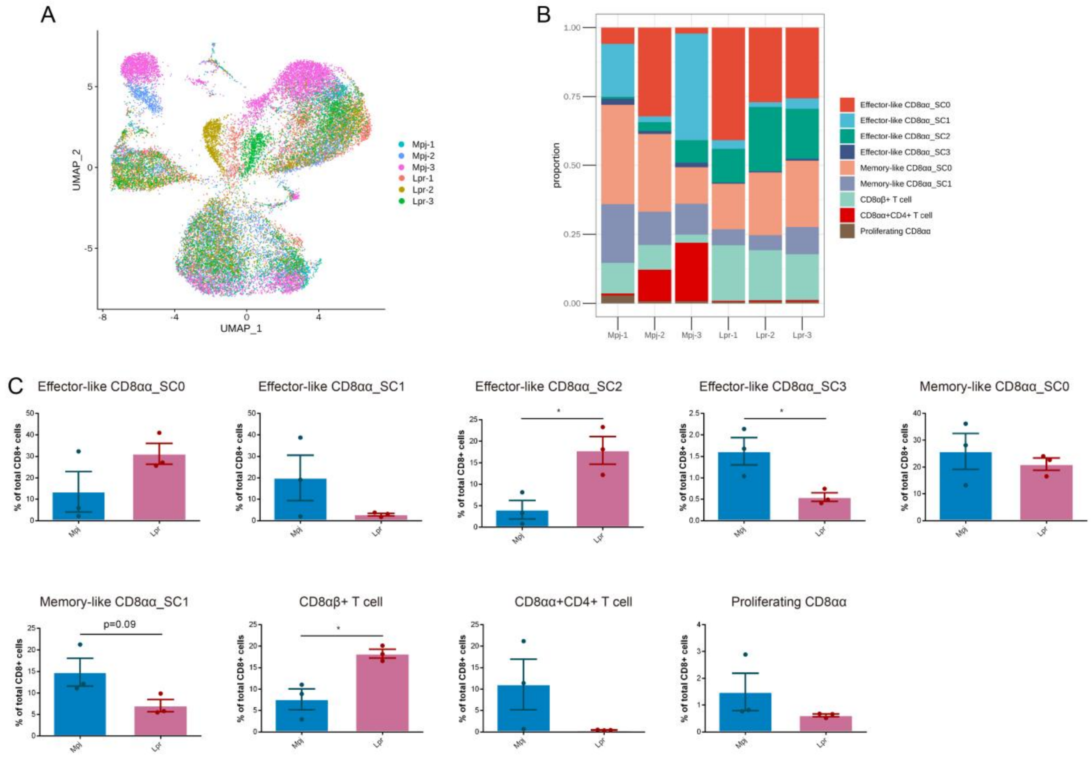

Supplementary Figure 4. scRNA-seq reveals altered composition of CD8+ IEL between Mpj and Lpr. (A) UMAP plots shows the distribution of CD8+ T cell cluster across various groups. (B) Bar plots depict the proportions of CD8+ T cell cluster across each individual sample. (C) Histogram plots illustrating the comparison of CD8+ T cell cluster between Mpj (n=3) and Lpr (n=3) based on scRNA-seq. The histograms for Mpj are presented in blue, histograms for Lpr are in red. Data were presented as mean ± SEM. *P < 0.05, **P < 0.01, ***P < 0.001, and ****P < 0.0001 by Student’s t test.

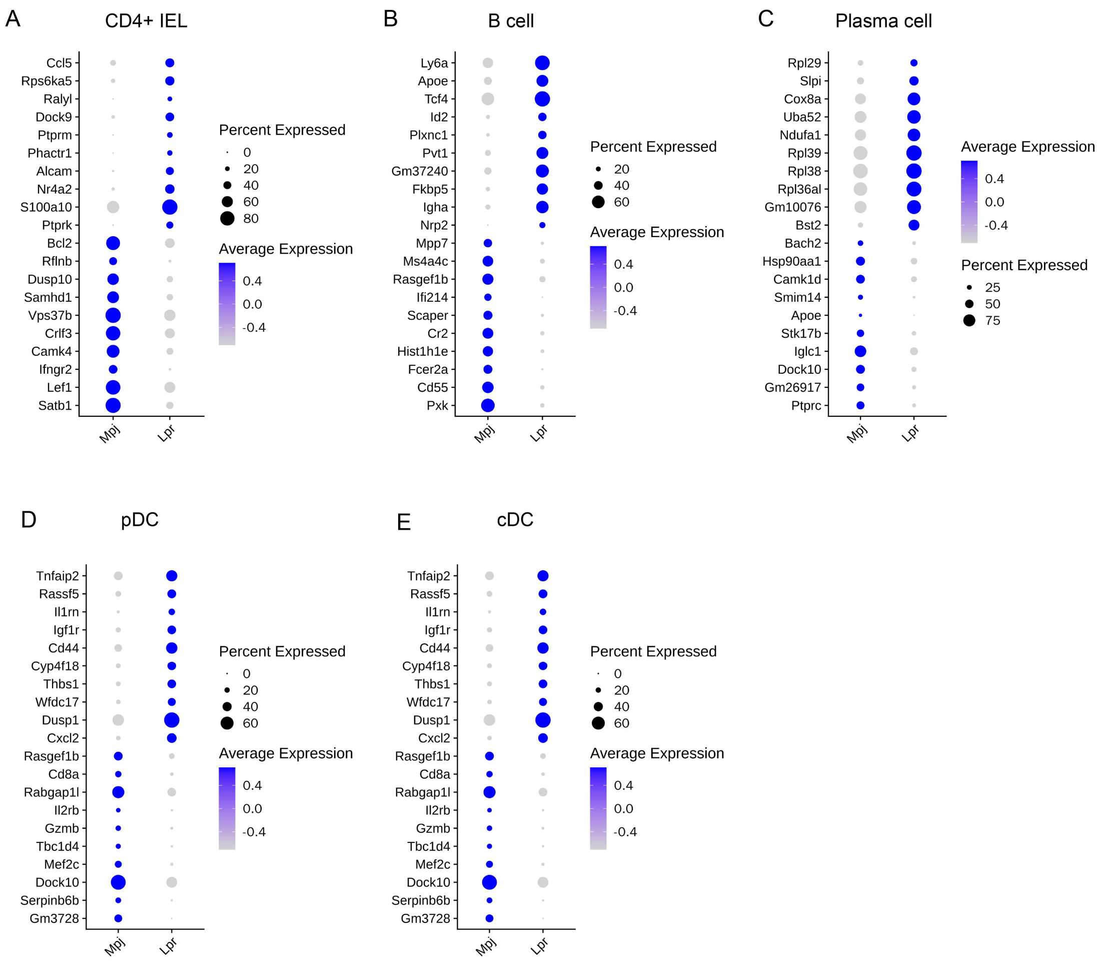

Supplementary Figure 5. DEGs for immune cells in intestinal epithelium layer across various groups. (A-E) Dot plot illustrating selected top 10 DEGs for CD4+IEL (A), B cell (B), Plasma cell (C), pDC (D), cDC (E) across various groups. Color saturation denotes the strength of gene expression on average, while the size of dots corresponds to the proportion of each cell cluster expressing the gene.

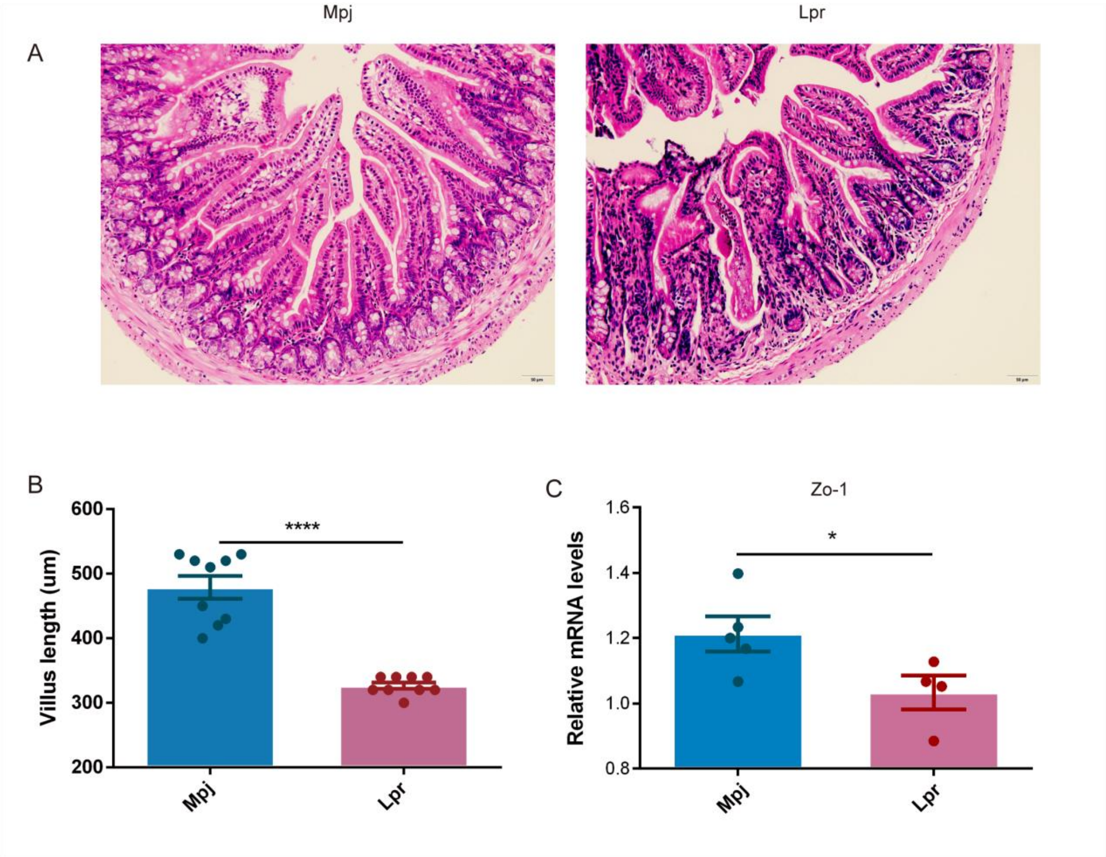

Supplementary Figure 6. Shorted villi and diminished expression of tight junction proteins were observed in lpr mice. (A) Representative images of H&E staining of ileum of Mpj and Lpr mice (Scale: 50um). (B) Histogram showing the comparison of villus length between Mpj and Lpr mice. (C) *Zo-1* expression in ileal tissues of Mpj and Lpr mice analyzed by qPCR. Data were presented as mean ± SEM. *P < 0.05, **P < 0.01, ***P < 0.001, and ****P < 0.0001 by Student's t test.

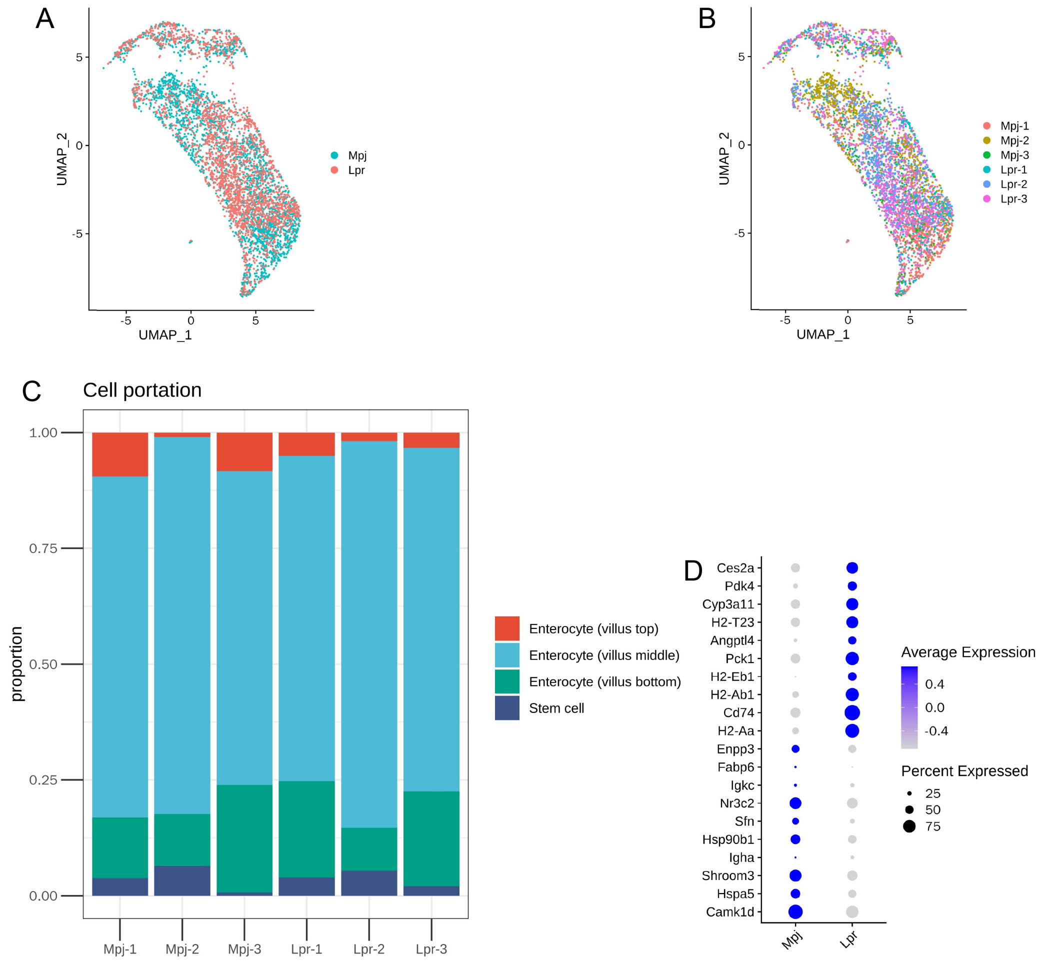

Supplementary Figure 7. scRNA-seq reveals the composition of enterocyte subsets between Mpj and Lpr. (A and B) UMAP plots shows the distribution of enterocyte subsets across various groups (A) and different individual samples (B). (C) Bar plots depict the proportions of enterocyte subsets across each individual sample. (D) Dot plot illustrating selected top 10 DEGs for all enterocytes across various groups. Color saturation denotes the strength of gene expression on average, while the size of dots corresponds to the proportion of each cell cluster expressing the gene.

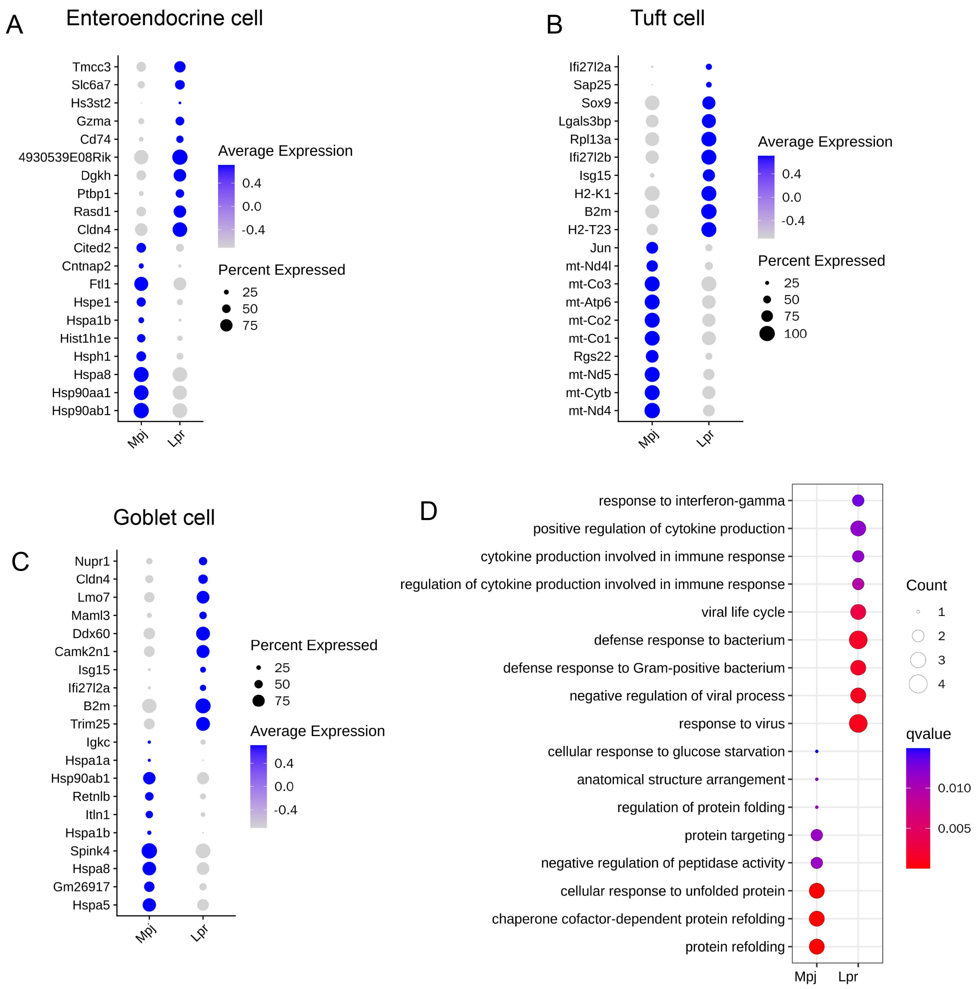

Supplementary Figure 8. DEGs for structural cells in intestinal epithelium layer across various groups. (A-C) Dot plot illustrating selected top 10 DEGs for enteroendocrine cell (A), tuft cell (B), and goblet cell (C) across various groups. Color saturation denotes the strength of gene expression on average, while the size of dots corresponds to the proportion of each cell cluster expressing the gene. (D) Bubble diagrams illustrate the Gene Ontology (GO) biological process (BP) terms enriched within goblet cell of Mpj and Lpr. Source data are provided as Supplementary Table 19.

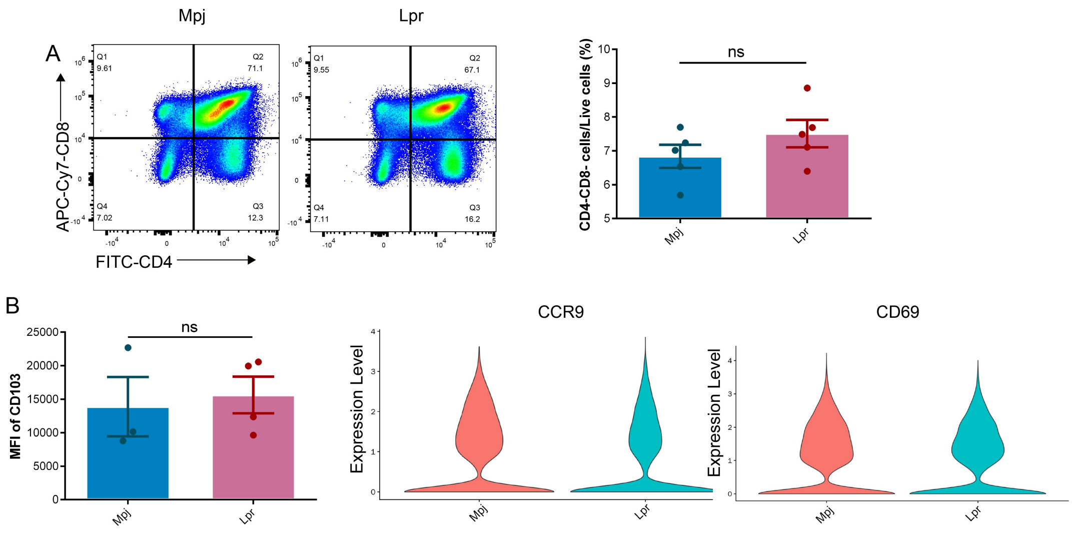

Supplementary Figure 9. The comparison of IEL development and γδT homing between Mpj and Lpr mice. (A) Representative flow cytometry analysis plot and quantification of CD4-CD8- thymocytes between Mpj and Lpr mice. (B) The marker associated with γδT homing between Mpj and Lpr mice. Data were presented as mean ± SEM. *P < 0.05, **P < 0.01, ***P < 0.001, and ****P < 0.0001 by Student's t test.
